# Copper Smelting Slag-Derived Fe_3_O_4_@Mesoporous Silica for Peroxymonosulfate Activation and Tetracycline Degradation: Performance, Mechanism, and Life Cycle Assessment

**DOI:** 10.3390/toxics14090757

**Published:** 2026-08-26

**Authors:** Changxin Li, Xiaoya Li, Jinyu Yang, Nan Liu, Shanpei Liu, Xianglong Huang, Huaxin Zhang

**Affiliations:** Hubei Key Laboratory of Drug Synthesis and Optimization, Jingchu University of Technology, Jingmen 448000, China; lxy301041@163.com (X.L.); 202412011@jcut.edu.cn (J.Y.); liunan@jcut.edu.cn (N.L.); jcutliusp@jcut.edu.cn (S.L.); jm_hxl@163.com (X.H.); h.x.zhang@yeah.net (H.Z.)

**Keywords:** copper smelting slag, tetracycline (TC) degradation, peroxymonosulfate (PMS) activation, mechanism, life cycle assessment

## Abstract

Tetracycline (TC) is a widely used antibiotic that is frequently detected in rivers, lakes and wastewater. Because TC is poorly removed by conventional biological treatment, its residues can harm aquatic organisms and promote the spread of antibiotic resistance; efficient and low-cost technologies for removing TC from water are therefore needed. In this study, copper smelting slag (CSS), an abundant industrial solid waste, was converted into a catalyst composed of Fe_3_O_4_ particles loaded on mesoporous silica (denoted Fe_3_O_4_@MS) via an alkali fusion–hydrothermal method. The catalyst was used to activate peroxymonosulfate (PMS), forming the Fe_3_O_4_@MS/PMS treatment system for the degradation of TC in aqueous solution. The effects of the main operating parameters (catalyst dosage, PMS concentration, initial pH and reaction temperature) on TC degradation were systematically evaluated. Under the optimized conditions (catalyst 0.5 g/L, PMS 1.0 mmol/L, initial pH 6.5, 25 °C), the Fe_3_O_4_@MS/PMS system removed 98.70% of 50 mg/L TC within 60 min. Radical quenching experiments and electron paramagnetic resonance (EPR) analysis revealed that TC was degraded through both radical pathways (hydroxyl •OH, sulfate SO_4_^•−^ and superoxide O_2_^•−^ radicals) and a non-radical pathway involving singlet oxygen (^1^O_2_), with •OH being the dominant reactive species. Nine degradation intermediates were identified by liquid chromatography–mass spectrometry (LC-MS), based on which three degradation pathways were proposed. Toxicity estimation indicated that ring-opening and deamination reactions are the key steps for detoxification. In addition, a life cycle assessment (LCA) across five selected impact categories identified the main environmental burdens associated with catalyst production. Overall, this work demonstrates that CSS-derived Fe_3_O_4_@MS is an efficient, low-cost and sustainable catalyst for PMS-based antibiotic removal from water, offering a circular-economy approach that couples solid-waste valorization with clean water production.

## 1. Introduction

The extensive application of antibiotics in human medicine, veterinary medicine, and aquaculture results in their persistent discharge into water bodies, thereby endangering both ecosystems and public health [1,2]. Tetracycline (TC), one of the most commonly prescribed antibiotics globally, is frequently detected in surface waters, groundwater, and wastewater treatment plant effluents [3,4,5,6]. The persistence of TC in the environment is primarily attributed to its stable aromatic structure and resistance to conventional biological treatment processes [7,8]. Moreover, the presence of residual TC in water bodies promotes the evolution and transmission of both resistant bacterial strains and the genetic determinants of antibiotic resistance, which have become a global health crisis [9,10,11]. Tetracycline is typically detected in surface water and groundwater at ng/L to low µg/ L levels, in municipal wastewater and hospital effluents at µg/L levels, and can reach tens to hundreds of µg/L (locally mg/L) in pharmaceutical production and livestock-farming wastewaters [12,13,14]. Even at these trace levels, TC exerts chronic ecotoxicological pressure on aquatic microorganisms and algae and, most critically, promotes the selection and dissemination of antibiotic-resistant bacteria and resistance genes. Thus, it is critically important to develop technologies that are both efficient and cost-effective for eliminating TC from water environments.

Owing to their generation of reactive oxygen species (ROS) with strong oxidation capacity, advanced oxidation processes (AOPs) have emerged as effective techniques for degrading persistent organic contaminants [15,16,17]. Among various AOPs, persulfate-based advanced oxidation processes have garnered increasing attention because of the advantages of sulfate radicals (SO_4_^•−^), including high oxidation potential (E^0^ = 2.5–3.1 V), long half-life (30–40 μs), and selective reactivity toward electron-rich organic compounds [18,19,20]. Multiple strategies can be employed to activate peroxymonosulfate (PMS, HSO_5_^−^), including heat, UV irradiation, transition metals, and carbon-based catalysts, to generate SO_4_^•−^ and hydroxyl radicals (•OH) [21,22,23,24]. Among these activation methods, heterogeneous transition metal catalysts offer advantages such as easy separation, reusability, and minimal secondary pollution [1,19,25,26].

Iron-based heterogeneous catalysts have been extensively investigated for PMS activation due to their abundance, environmental compatibility, and high catalytic activity [27,28,29,30]. Iron oxides, particularly magnetite (Fe_3_O_4_), have shown promising performance in activating PMS for pollutant degradation due to the presence of both Fe^2+^ and Fe^3+^ species, which facilitate efficient redox cycling [19,29,31]. However, the practical application of Fe_3_O_4_ nanoparticles is often hindered by issues such as aggregation, limited surface area, and potential iron leaching, which can lead to reduced catalytic activity and secondary contamination. To address these challenges, various support materials, including mesoporous silica, have been employed to immobilize Fe_3_O_4_ nanoparticles, providing enhanced dispersion, increased surface area, and improved stability [32,33,34,35].

In recent years, the utilization of industrial solid wastes as raw materials for catalyst synthesis has gained significant attention due to its economic and environmental benefits [36,37,38,39]. Copper smelting slag (CSS), a byproduct of copper extraction processes, is generated in large quantities worldwide and contains substantial amounts of iron and silicon oxides [40,41]. Disposing of CSS consumes extensive land resources and simultaneously threatens the environment through heavy metal leaching. The valorization of CSS into functional materials represents a sustainable approach to waste management and resource recovery. Several studies have reported the extraction of valuable components from CSS for the synthesis of various materials, including adsorbents and catalysts [42,43,44]. However, the application of CSS-derived materials for PMS activation in antibiotic degradation remains largely unexplored.

Herein, we report the synthesis of CSS-derived Fe_3_O_4_@mesoporous silica (Fe_3_O_4_@MS) via an alkali fusion–hydrothermal method and its application as a heterogeneous catalyst for PMS activation to degrade TC in aquatic environments. The synthesis strategy involves the extraction of silicon from CSS to form a mesoporous silica support, followed by in-situ growth of Fe_3_O_4_ nanoparticles within the mesoporous structure. The obtained Fe_3_O_4_@MS composite was comprehensively characterized using various techniques to elucidate its structural, textural and surface chemical. The catalytic performance of Fe_3_O_4_@MS for TC degradation was systematically evaluated, including the effects of key operational parameters such as catalyst dosage, PMS concentration, initial TC concentration, temperature, pH, and coexisting anions. The degradation kinetics were investigated in detail under various operational parameters, namely catalyst dosage, PMS dosage, initial solution pH, initial TC concentration and reaction temperature. Consecutive degradation cycles were used to examine the catalyst’s recyclability and stability, and the mechanisms of PMS activation and TC degradation were elucidated through radical quenching experiments, EPR spectroscopy, and LC-MS analysis. Finally, based on the identified intermediates, plausible degradation pathways and toxicity analysis were proposed. Moreover, a thorough evaluation of the environmental impact and life cycle assessment (LCA) associated with the synthesis, application, and disposal of the Fe_3_O_4_@MS catalyst, highlighting its potential as a sustainable solution for antibiotic wastewater treatment.

## 2. Materials and Methods

### 2.1. Experimental Materials

In this study, copper smelting slag (CSS) was obtained from a copper smelting enterprise in Huangshi City, Hubei Province, China. Prior to use, the raw CSS was crushed, ground, and sieved through a 200-mesh screen, followed by drying at 100 °C for 2 h. The major oxide components in CSS are Fe_2_O_3_ (62.02 wt%), SiO_2_ (23.85 wt%), Al_2_O_3_ (5.04 wt%), and CaO (3.95 wt%), with lesser amounts of ZnO (2.96 wt%) and MgO (0.43 wt%). Tetracycline (TC, C_22_H_24_N_2_O_8_, ≥99%) and potassium peroxymonosulfate (PMS, 2KHSO_5_·KHSO_4_·K_2_SO_4_) were purchased from Aladdin Industrial Corporation (Shanghai, China). Sodium hydroxide (NaOH), sulfuric acid (H_2_SO_4_), absolute ethanol (C_2_H_5_OH) and polyethylene-polypropylene glycol (P123) were procured from Sinopharm Chemical Reagent Co., Ltd. (Shanghai, China). Tert-butyl alcohol (TBA, C_4_H_10_O, ≥99.5%), methanol (MeOH, ≥99.9%), L-histidine (L-His, ≥99%) and p-benzoquinone (p-BQ, C_6_H_4_O_2_, ≥99%) were purchased from Macklin Biochemical Technology Co., Ltd. (Shanghai, China) and employed as radical scavengers in quenching experiments. Analytical-grade chemical reagents were utilized without further purification. Deionized (DI) water was employed for all solution preparation and rinsing steps in the experiments.

### 2.2. Preparation Process of Fe_3_O_4_@MS

The Fe_3_O_4_@MS was prepared via a two-stage procedure involving alkali fusion pretreatment of CSS followed by a hydrothermal method. Initially, 5.0 g of the pretreated CSS (as described in Section 2.1) was thoroughly mixed with 6.5 g of NaOH solid (CSS: NaOH mass ratio of 1:1.3) in an agate mortar. The mixture was thereafter loaded into a nickel crucible and calcined in a muffle furnace at 600 °C for 1.0 h. The obtained fused product, which contained soluble sodium silicate, was left to cool to ambient temperature. Subsequently, the 10 g cooled fused product was introduced 100 mL of DI water while stirring vigorously at 100 °C for 1.0 h to facilitate the leaching of silicate species. Then, 0.05 g P123 was added to the aforementioned reaction system and the mixture’s pH was gradually brought to around 5.0 with 2 mol/L H_2_SO_4_ solution. The resulting suspension was placed in a water bath for hydrothermal treatment at 100 °C for 2.0 h. After cooling to room temperature, the precipitate was collected by filtration, washed repeatedly with deionized water and absolute ethanol until the supernatant reached neutral pH, and then dried at 80 °C overnight. The obtained dark brown powder, designated Fe_3_O_4_@MS, was kept in a desiccator for subsequent characterization and use.

### 2.3. Degradation Procedures of TC

All batch degradation experiments were conducted in 250 mL conical flasks placed in a thermostatic shaker (25 °C) at a stirring speed of 200 rpm to ensure uniform mixing. Unless otherwise specified, the reaction system contained 100 mL of TC solution with an initial concentration of 50 mg/L, 0.5 g/L of Fe_3_O_4_@MS catalyst, and 1.0 mmol/L of PMS. To initiate the reaction, PMS was added to the suspension containing both the catalyst and TC. At predetermined time points (0–60 min: 0, 1, 3, 5, 10, 15, 20, 30, 40, 50, 60), 2 mL aliquots were withdrawn, rapidly filtered (0.22 μm nylon membrane), and mixed with 0.5 mL of methanol to stop any radical reactions. The residual TC level in the filtrate was then quantified via UV–vis spectrophotometry (752N, INESA, Shanghai, China) at 357 nm. A calibration curve (R^2^ > 0.999) was established prior to measurement.

To assess the degradation kinetics, the pseudo-first-order model (−ln(C_0_/C_t_) = k_obs_ × t) was fitted to the experimental data, with C_0_ and C_t_ being the TC concentrations at time 0 and time t, and k_obs_ is the observed rate constant (min^−1^).

A systematic investigation was conducted into how TC degradation depends on several operational parameters: catalyst dosage (0.1–0.7 g/L), PMS concentration (0.2–1.5 mmol/L), starting TC concentration (10–50 mg/L), initial pH (3.0–13.0, using 1.0 mol/L H_2_SO_4_ or NaOH), temperature (20–35 °C), and coexisting anions (Cl^−^, HCO_3_^−^, NO_3_^−^, SO_4_^2−^, H_2_PO_4_^−^). TC is an amphoteric molecule with three acid dissociation constants (pKa1 ≈ 3.3, pKa2 ≈ 7.7, pKa3 ≈ 9.7), so it occurs in water as a cationic (H_3_TC^+^), neutral/zwitterionic (H_2_TC), or anionic (HTC^−^/TC^2−^) species depending on pH.

### 2.4. Radical Quenching Experiments

Identification of the dominant ROS involved in the Fe_3_O_4_@MS/PMS system was performed by radical quenching experiments. Various scavengers were added to the reaction solution prior to the addition of PMS: tert-butyl alcohol (TBA, 100 mmol/L) for hydroxyl radicals (•OH), methanol (MeOH, 100 mmol/L) for both •OH and sulfate radicals (SO_4_^•−^), p-benzoquinone (p-BQ, 10 mmol/L) for superoxide radicals (O_2_^•−^), and L-histidine (10 mmol/L) for singlet oxygen (^1^O_2_). The change in TC removal efficiency was compared with the control without scavenger.

### 2.5. Reusability and Stability Test Procedure of Fe_3_O_4_@MS

Reusability and stability of Fe_3_O_4_@MS were evaluated through consecutive degradation cycles under the same conditions. Following each degradation cycle, the catalyst was recovered by filtration, rinsed with deionized water and ethanol, dried at 60 °C overnight, and then reutilized in the subsequent cycle. The leached iron ion concentration in the post-reaction solution was measured using an atomic absorption spectrometer (CAAM-2001C, HTH Instrument Co., Ltd., Beijing, China).

### 2.6. Life Cycle Assessment Methodology

#### 2.6.1. Goal and Scope Definition

**Figure 1:**
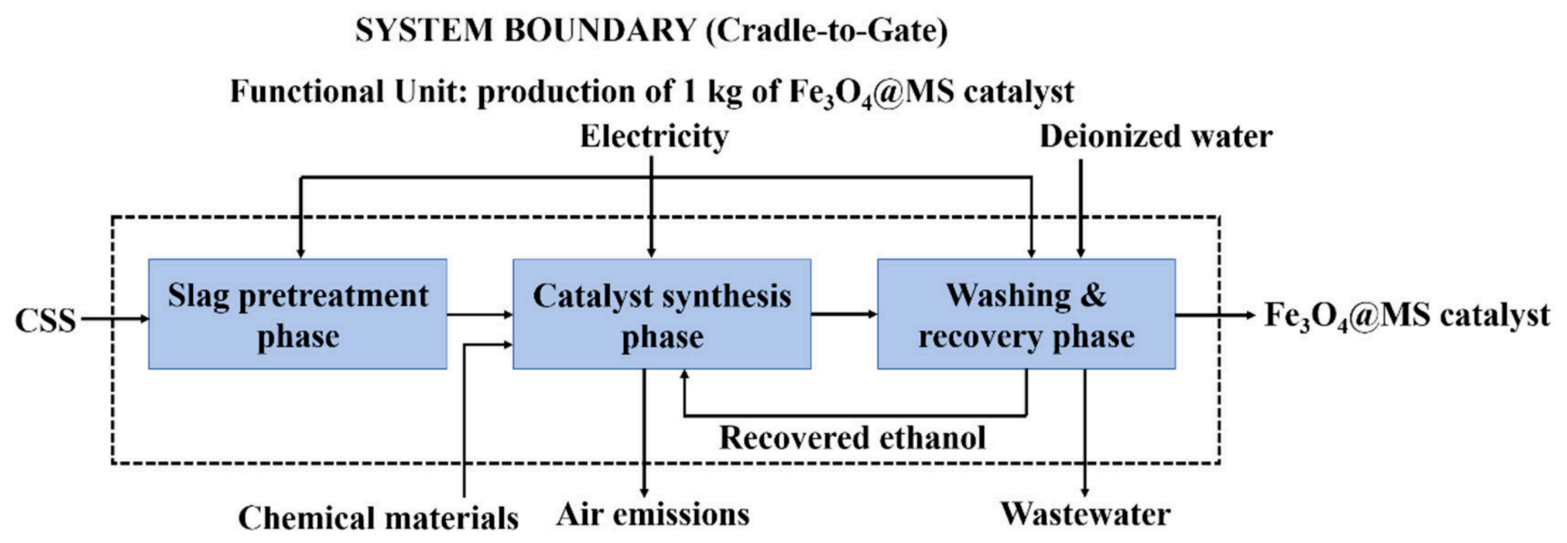
Cradle-to-gate system boundary for the synthesis of Fe_3_O_4_@MS catalyst from CSS.

The primary objective of this LCA is to assess the environmental impact of producing the Fe_3_O_4_@MS composite using CSS as a feedstock. According to ISO 14040/14044 [45,46], the functional unit (FU) is defined as 1 kg of the synthesized catalyst. As illustrated in Figure 1, the system boundary is defined as “cradle-to-gate”, encompassing three main stages: (1) slag pretreatment phase, (2) catalyst synthesis phase, and (3) washing and recovery phase. Notably, a cut-off approach is applied to the CSS; as an industrial solid waste, it enters the system burden-free, with only its subsequent transportation and processing impacts considered. Furthermore, an internal solvent recycling loop using recovered ethanol is integrated into the boundary to reflect the actual sustainable engineering design.

#### 2.6.2. Life Cycle Inventory (LCI) and Impact Assessment (LCIA)

Primary data for the foreground system were collected from laboratory-scale experiments and normalized to the functional unit. This included the consumption of deionized water, chemical materials such as ethanol and sodium hydroxide, and direct emissions to air and wastewater. To avoid the extreme overestimation of energy consumption inherent in laboratory-scale equipment, electricity consumption was estimated at a conservative industrial benchmark of 20 kWh/kg of catalyst. This adjustment accounts for the significantly higher thermal efficiency of large-scale industrial reactors compared to laboratory muffle furnaces. The comprehensive life cycle inventory is tabulated in Table S1 of the Supplementary Materials. Background data for the production of upstream materials and the Chinese medium-voltage electricity market were sourced from the Ecoinvent 3 database within the openLCA software. The environmental impact analysis was performed employing the ReCiPe 2016 Midpoint H method. To comprehensively understand the environmental hotspots, five key impact categories were selected for contribution analysis: Global Warming Potential (GWP), Fossil Resource Scarcity (FRS), Land Use (LU), Freshwater Ecotoxicity Potential (FETP), and Mineral Resource Scarcity (MRS). For clarity in LCI modeling and visualization, inputs with minor contributions such as sulfuric acid and transport were aggregated into an Others category.

### 2.7. Characterizations

The as-synthesized Fe_3_O_4_@MS composite was examined by field emission scanning electron microscopy (SEM, Zeiss Sigma 500, Carl Zeiss Microscopy, Oberkochen, Germany) equipped with an EDS detector for elemental mapping to reveal its morphology and microstructure. X-ray diffraction (XRD) was employed to identify the crystalline phases and structural properties, using a Bruker D8 Advance diffractometer (Bruker AXS, Karlsruhe, Germany) with Cu Kα radiation (λ = 1.5406 Å) over 2θ = 1–80° at 2°/min. The oxidation states and surface chemical composition of the elements were analyzed by X-ray photoelectron spectroscopy (XPS) on a Thermo Scientific ESCALAB Xi+ spectrometer (Thermo Fisher Scientific, Waltham, MA, USA) with monochromatic Al Kα radiation (hν = 1486.6 eV). All binding energies were calibrated using the adventitious carbon C 1s peak at 284.8 eV as reference. Electron paramagnetic resonance (EPR) spectroscopy was conducted using a Bruker A300 emxplus spectrometer (Bruker Corporation, Billerica, MA, USA) at room temperature. Nitrogen adsorption–desorption isotherms (77 K, Micromeritics ASAP 2460, Micromeritics Instrument Corporation, Norcross, GA, USA) were employed to characterize the textural properties (specific surface area, pore volume, and pore size distribution). After degassing at 200 °C under vacuum for 6 h, the BET method was applied to determine the specific surface area, and the BJH model was used to derive the pore size distribution from the desorption branch. The point of zero charge (pH_pzc_) of Fe_3_O_4_@MS was determined by measuring zeta potential over pH 2.0–14.0 (Zetasizer Nano ZS90, Malvern Instruments Ltd., Worcestershire, UK). Samples were dispersed in DI water, with pH adjusted using 1.0 M HCl or NaOH. LC-MS analysis ( LCMS-2020, Shimadzu Corporation, Kyoto, Japan) was performed to identify degradation intermediates. Chromatographic separation was on a ShimNex UP C18 column (50 × 4.6 mm, 5 µm) (Shimadzu (Shanghai) Global Laboratory Consumables Co., Ltd., Shanghai, China) at 40 °C, with a mobile phase of (A) 0.1% formic acid in water and (B) 0.1% formic acid in acetonitrile under gradient elution at 2.0 mL/min. The mass spectrometer was performed using an ESI source with polarity switching in both positive and negative ion modes for intermediate identification. Ion chromatography (IC) analysis was performed using an IC-6000 ion chromatograph (Anhui Wayeal Science and Technology Co., Ltd., Hefei, China). The carbonate/bicarbonate-based eluent is delivered at a flow rate of 1.0 mL/min, the injection volume is typically 25 μL, and the column oven temperature is maintained at 30 °C. Prior to analysis, water samples are first filtered through a 0.22 μm membrane filter and, if necessary, diluted with deionized water to bring the nitrate concentration within the calibration range.

## 3. Results and Discussion

### 3.1. Characterization of Fe_3_O_4_@MS

The crystalline structure of the materials was investigated by XRD. As presented in Figure S1, the XRD pattern of Fe_3_O_4_@MS is defined by its absence of sharp peaks and presence of a characteristic broad, diffuse hump rather than sharp crystalline peaks. This primary diagnostic feature reflects the continuous random network structure and serves as a fingerprint for amorphous phase identification. Moreover, the Fe_3_O_4_@MS composite displayed additional diffraction peaks at 2θ = 30.2°, 35.5°, 43.1°, 53.5°, 57.0°, and 62.6°, corresponding to the (220), (311), (400), (422), (511), and (440) planes of cubic Fe_3_O_4_ (JCPDS No. 19-0629), respectively [47,48,49]. The absence of characteristic peaks from other iron oxides (e.g., α-Fe_2_O_3_ or γ-Fe_2_O_3_) confirmed the phase purity of the magnetite. SEM was then used to examine the morphology and microstructure of the as-synthesized Fe_3_O_4_@MS composite. As shown in Figure S2, the Fe_3_O_4_@MS exhibited irregular spherical particles with a relatively uniform size distribution. The mesoporous silica matrix presented a typical wormhole-like porous structure, while the iron oxide nanoparticles (appearing as darker contrast) were evenly distributed across both the external surface and the internal pores of the silica matrix. Energy dispersive spectroscopy (EDS) mapping analysis (Figure S3) revealed that Si, O, and Fe were uniformly dispersed across the composite, indicating that iron species were well-dispersed on the mesoporous silica support without significant aggregation. N_2_ adsorption–desorption analysis confirmed that the Fe_3_O_4_@MS composite derived from CSS possesses well-developed mesoporosity with a type IV isotherm and an H3-type hysteresis loop (see Figure S4). Measurements yielded a BET specific surface area of 56.49 m^2^/g, a total pore volume of 0.24 cm^3^/g, and a BJH desorption average pore diameter of about 18.11 nm.

### 3.2. Efficiency of TC Degradation During Different Reaction Processes

**Figure 2:**
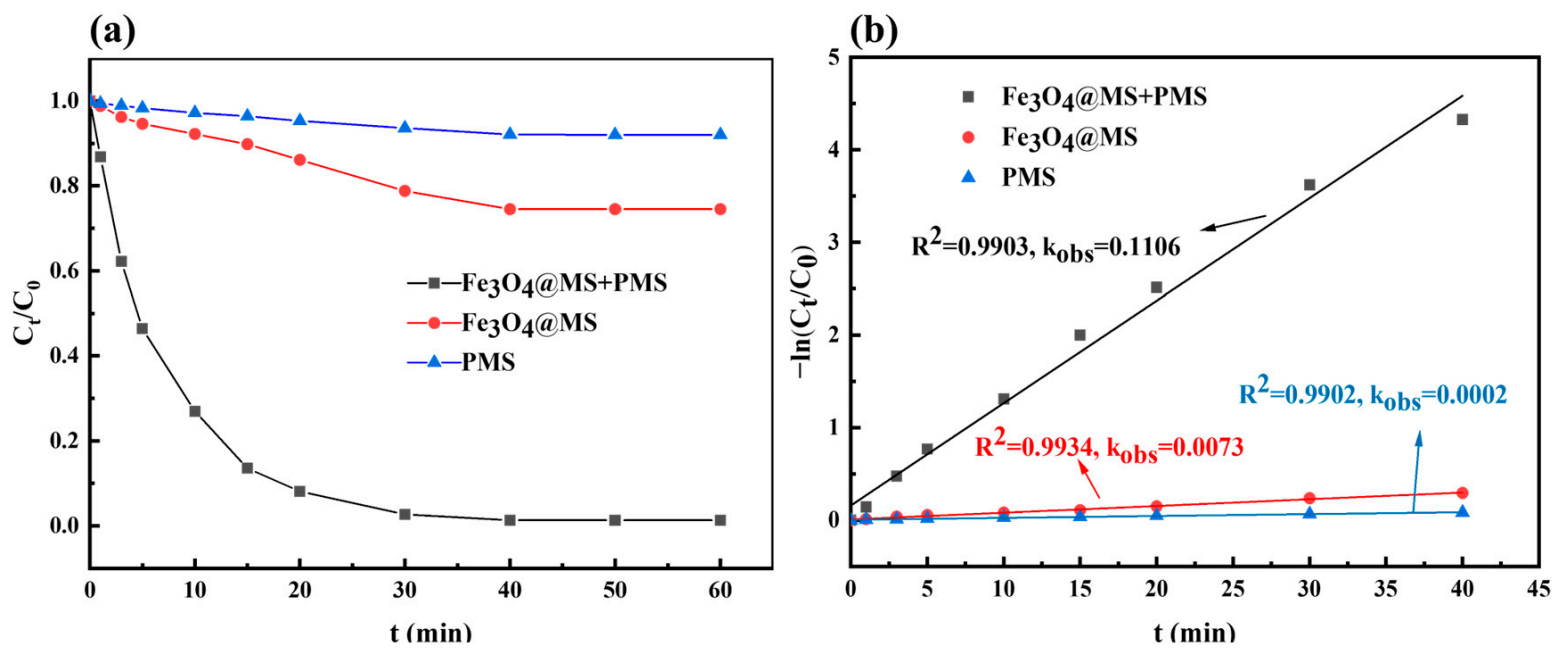
(**a**) TC degradation and (**b**) calculated first-order kinetic constants under different catalytic degradation systems (Experimental conditions: TC concentration = 50 mg/L, catalyst dosage = 0.5 g/L, PMS concentration = 1.0 mmol/L, initial pH = 6.8, temperature = 25 °C).

To evaluate the catalytic performance of the Fe_3_O_4_@MS and elucidate the synergistic effect between the catalyst and PMS, comparative degradation experiments of TC were conducted under identical conditions (TC concentration = 50 mg/L, catalyst dosage = 0.5 g/L, PMS concentration = 1.0 mmol/L, initial pH = 6.8, temperature = 25 °C) across three different reaction systems: (I) PMS alone, (II) Fe_3_O_4_@MS alone (without PMS), and (III) Fe_3_O_4_@MS/PMS system. The temporal profile of TC removal efficiency over 60 min is displayed in Figure 2 under different reaction systems.

As depicted in Figure 2a, the degradation of TC was negligible in the system containing only PMS, with less than 8% removal after 60 min of reaction. This observation indicates that PMS alone, without an appropriate activator, exhibits limited oxidative capability toward TC under ambient conditions, consistent with previous reports on the low self-decomposition rate of PMS in the absence of catalysts. In contrast, the Fe_3_O_4_@MS alone system (without PMS) exhibited obvious enhanced TC removal (approximately 29% after 60 min) compared to the PMS alone system. This increase may be attributed to the adsorption of TC molecules onto the mesoporous silica surface and the additional contribution of surface complexation between TC molecules and the dispersed Fe_3_O_4_ nanoparticles, as well as the possible generation of trace ROS from dissolved oxygen under ambient conditions. However, the removal efficiency remained limited, confirming that physical adsorption and surface interactions alone cannot achieve satisfactory TC elimination. Remarkably, the Fe_3_O_4_@MS/PMS system demonstrated significantly superior performance, achieving a TC degradation efficiency of 98.7% within 60 min of reaction. More importantly, the degradation process exhibited a rapid initial phase, with approximately 92% of TC removed within the first 20 min, followed by a slower removal phase. This biphasic behavior is characteristic of heterogeneous Fenton-like reactions, where the fast initial stage corresponds to the rapid generation of ROS from PMS activation by surface Fe^2+^/Fe^3+^ redox cycling, while the subsequent slower stage may be attributed to the gradual depletion of active sites and consumption of PMS [47,48,49,50].

To enable a quantitative comparison of degradation rates across different systems, the experimental data were fitted to the pseudo-first-order model. The corresponding kinetic plots (−ln(C_t_/C_0_) vs. time) and calculated rate constants (k_obs_) are presented in Figure 2b. The Fe_3_O_4_@MS/PMS system yielded the maximum apparent rate constant (k_obs_ = 0.1107 min^−1^), which was approximately 15.2 times higher than that of Fe_3_O_4_@MS alone (k_obs_ = 0.0073 min^−1^) and more than 500-fold higher than that observed for PMS alone (k_obs_ = 0.0002 min^−1^). The high correlation coefficients (R^2^ > 0.99) confirmed that the pseudo-first-order kinetic model adequately described the degradation process. Furthermore, the synergistic coefficient (SC) was calculated based on the kinetic rate constants to quantify the synergistic effect between Fe_3_O_4_@MS and PMS. The SC value was determined to be 14.75, indicating a strong synergistic interaction that significantly enhances the overall degradation performance beyond the sum of individual contributions. To benchmark the catalytic performance of the Fe_3_O_4_@MS, a comprehensive comparison was conducted with previously reported heterogeneous catalysts for PMS activation toward TC degradation. A summary of the TC degradation efficiency under different heterogeneous catalysts for PMS activation is presented in Table S2. The Fe_3_O_4_@MS catalyst derived from CSS exhibits a 98.70% for TC degradation under ambient conditions, which is competitive with many reported catalysts.

To further verify the essential role of Fe_3_O_4_ nanoparticles in PMS activation, a control experiment was conducted using Fe^3+^ ions (1.0 mmol/L) as a homogeneous catalyst under otherwise identical conditions. The Fe^3+^/PMS system achieved approximately 56.56% TC degradation within 60 min (k_obs_ = 0.0146 min^−1^), which was substantially lower than in the heterogeneous Fe_3_O_4_@MS/PMS system. This difference highlights the advantages of the heterogeneous catalyst, including the confinement effect provided by the mesoporous structure, the enhanced stability of iron species, and the continuous exposure of active sites for sustained PMS activation. Additionally, the mineralization efficiency of TC was evaluated by measuring total organic carbon (TOC) removal after 60 min of reaction. The Fe_3_O_4_@MS/PMS system achieved a TOC removal efficiency of 61.35%, indicating that a substantial portion of TC was not only transformed but also completely mineralized into CO_2_ and H_2_O. In contrast, the PMS alone and Fe_3_O_4_@MS alone systems exhibited TOC removal efficiencies of only 2.5% and 1.3%, respectively, further confirming the superior oxidative capability of the combined system. These comparative results unequivocally demonstrate that the Fe_3_O_4_@MS composite synthesized from CSS functions as a highly efficient heterogeneous material for PMS activation, and the synergistic combination of mesoporous silica support and iron oxide nanoparticles plays a crucial role in achieving high-performance degradation of TC.

### 3.3. TC Degradation Kinetics by Fe_3_O_4_@MS

#### 3.3.1. Effect of Catalyst Dosage

**Figure 3:**
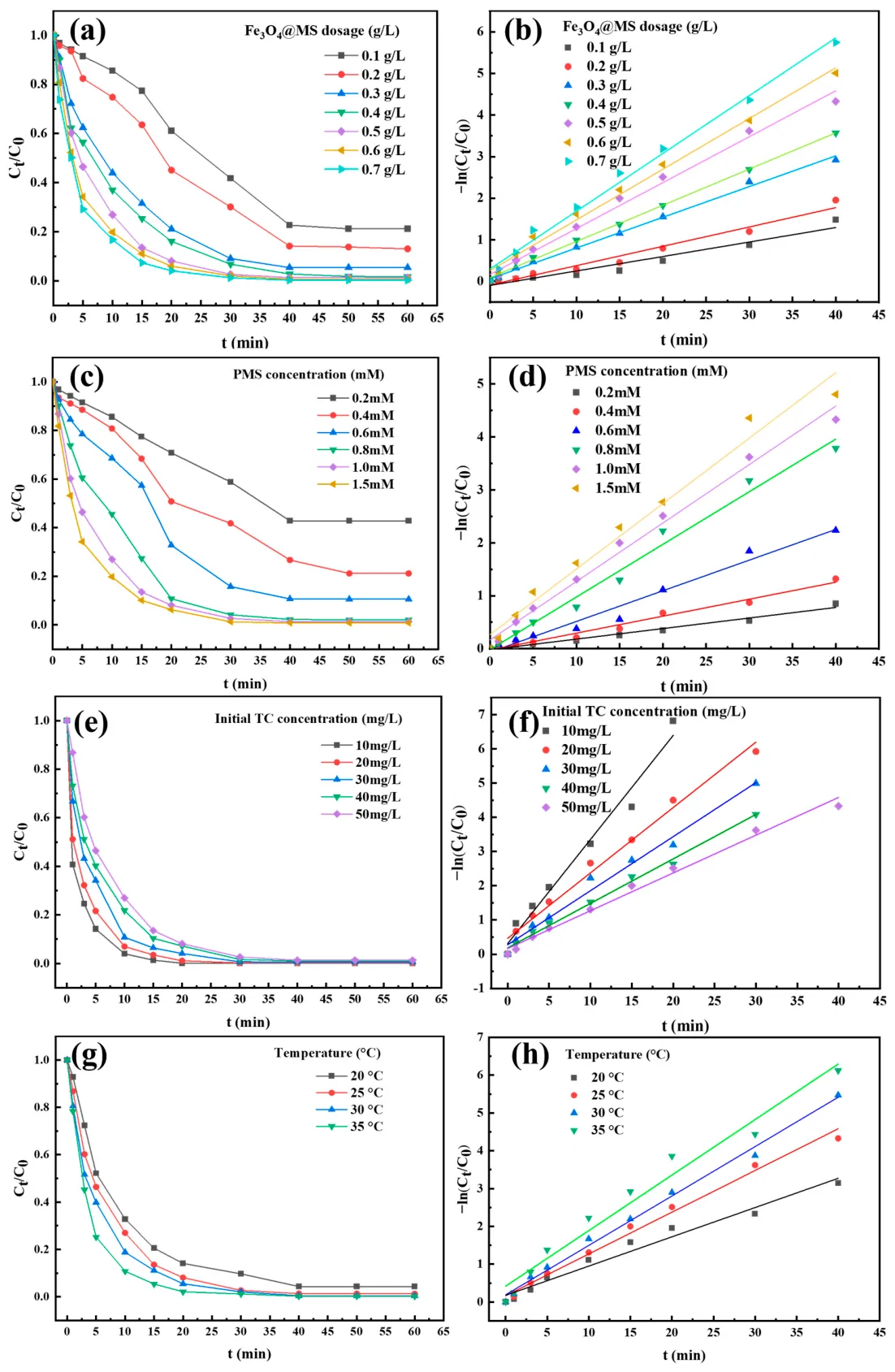
(**a**) TC degradation and (**b**) kinetic fitting under different catalyst dosages (Experimental conditions: TC concentration = 50 mg/L, PMS concentration = 1.0 mmol/L, initial pH = 6.8, temperature = 25 °C); (**c**) TC degradation and (**d**) kinetic fitting under different PMS concentrations (Experimental conditions: TC concentration = 50 mg/L, catalyst dosage = 0.5 g/L, initial pH = 6.8, temperature = 25 °C); (**e**) TC degradation and (**f**) kinetic fitting under different initial TC concentrations (Experimental conditions: PMS concentration = 1.0 mmol/L, catalyst dosage = 0.5 g/L, initial pH = 6.8, temperature = 25 °C); (**g**) TC degradation and (**h**) kinetic fitting under different temperatures (Experimental conditions: TC concentration = 50 mg/L, PMS concentration = 1.0 mmol/L, catalyst dosage = 0.5 g/L, initial pH = 6.8).

To study how Fe_3_O_4_@MS concentration affects TC degradation efficiency and kinetics, batch experiments were carried out over a dosage range of 0.1–0.7 g/L, while keeping other operational parameters constant (TC concentration = 50 mg/L, PMS concentration = 1.0 mmol/L, initial pH = 6.8, temperature = 25 °C). Figure 3 presents the temporal profiles of TC degradation and the associated pseudo-first-order kinetic fits. Table S3 summarizes the kinetic parameters.

Figure 3a shows that TC degradation efficiency strongly depended on the Fe_3_O_4_@MS dosage. Raising the catalyst dosage from 0.1 to 0.5 g/L significantly enhanced the 60-min TC removal efficiency from 78.80% to 98.69%. The improvement is due to a greater number of available Fe^2+^/Fe^3+^ active sites on the catalyst surface, which facilitate the generation of more ROS through PMS activation. With a higher catalyst dosage, a greater number of Fe_3_O_4_ nanoparticles are accessible for the redox cycling between Fe^2+^ and Fe^3+^, thereby accelerating the production of sulfate radicals (SO_4_^•−^), hydroxyl radicals (•OH), and other ROS responsible for TC oxidation [28,51,52]. However, further increasing the catalyst dosage to 0.6 g/L and 0.7 g/L resulted in only marginal improvements in degradation efficiency, achieving 99.33% and 99.68% TC removal, respectively, after 60 min. This observation suggests that beyond an optimal catalyst dosage (0.5 g/L), the degradation process becomes limited by other factors such as PMS concentration or mass transfer constraints rather than the number of active sites. Excessive catalyst loading may induce agglomeration, thereby diminishing the effective surface area and disrupting the homogeneous dispersion of Fe_3_O_4_ nanoparticles. Moreover, an excessively high catalyst dosage could potentially induce radical scavenging effects, where excessive Fe^2+^ species react with generated SO_4_^•−^ to form less reactive Fe^3+^ and SO_4_^2−^, thereby diminishing the overall oxidation efficiency [50,52,53].

To quantitatively describe the relationship between catalyst dosage and degradation kinetics, the experimental data were fitted to an empirical power-law relationship, as expressed in Equation (1), where k is the empirical rate constant, [*Catalyst*] is the catalyst concentration (g/L), and α is the reaction order with respect to the catalyst dosage.

(1) $$k_{\mathrm{obs}}=k\times {\left[Catalyst\right]}^{\alpha }$$

From the kinetic analysis of catalyst dosage effects (see Figure 3b and Table S3), the relationship between k_obs_ and catalyst dosage was established. A double-logarithmic plot of ln(k_obs_) versus ln[Catalyst] (see Figure S5) yielded a straight line with a slope of 0.7433 (R^2^ = 0.9809) within the catalyst dosage range of 0.1–0.7 g/L. Thus, the reaction order with respect to catalyst dosage was determined to be 0.7433. This fractional order indicates that the catalytic activation of PMS is not linearly proportional to the catalyst loading, likely due to partial utilization of active sites and mass transfer limitations at higher catalyst concentrations. The positive correlation confirms that higher catalyst loadings provide more active sites for PMS activation, generating greater quantities of ROS for TC degradation. However, the diminishing increase in k_obs_ at dosages beyond 0.5 g/L suggests approaching saturation of active sites or possible mass transfer limitations.

#### 3.3.2. Effect of PMS Concentration

To investigate the effect of PMS concentration (0.2–1.5 mmol/L) on TC degradation kinetics, batch experiments were performed under constant conditions (TC concentration = 50 mg/L, catalyst dosage = 0.5 g/L, initial pH = 6.8, temperature = 25 °C). Figure 3c shows a strong positive correlation between TC degradation efficiency and PMS concentration. Raising the PMS level from 0.2 to 1.0 mmol/L and then to 1.5 mmol/L increased the 60 min TC removal efficiency from 57.20% to 98.69% and 99.18%, respectively. This improvement results from greater PMS availability, which enhances collision frequency with surface Fe^2+^/Fe^3+^ sites on Fe_3_O_4_@MS, thus boosting ROS generation [47,54,55].

The degradation kinetics at different PMS concentrations were analyzed using the pseudo-first-order kinetic model. As shown in Figure 3d, the linear relationships between −ln(C_0_/C_t_) and reaction time (R^2^ > 0.97) confirmed the applicability of this model across all investigated PMS concentrations and the derived rate constants are presented in Table S4. To quantitatively describe the dependence of the degradation rate on PMS concentration, the experimental data were fitted to a power-law expression. The double-logarithmic plot of ln(k_obs_) versus ln[PMS] (Figure S6) exhibited high linearity (R^2^ = 0.9739) within the 0.2–1.5 mmol/L PMS concentration range. The slope of the regression line yielded a reaction order (β) of 1.0149 with respect to PMS concentration, which is approximately unity. This near-first-order behavior implies that the degradation rate is directly proportional to PMS concentration under the tested conditions, which in turn suggests a rate-determining step involving the interaction of a single PMS molecule with an active site on the catalyst surface. Namely, the initial step in the activation process involves the adsorption of HSO_5_^−^ onto the Fe^2+^ active sites on the Fe_3_O_4_@MS surface. The surface-mediated electron transfer from Fe^2+^ to the adsorbed HSO_5_^−^ is the rate-limiting step for ROS generation.

#### 3.3.3. Effect of Initial TC Concentration

To examine the effect of initial TC concentration (10–50 mg/L) on degradation kinetics, batch experiments were performed under otherwise constant conditions (catalyst dosage = 0.5 g/L, PMS concentration = 1.0 mmol/L, initial pH = 6.8, temperature = 25 °C). Figure 3e,f and Table S5 present the degradation profiles, kinetic fitting, and calculated parameters. As shown in Figure 3e, TC degradation efficiency did not significantly decline with rising initial TC concentration. At 10 mg/L TC, the efficiency reached 99.89% after 60 min; even at 50 mg/L, it remained as high as 98.69%. The above indicates that the Fe_3_O_4_@MS exhibits good catalytic performance across various concentrations. The minor reduction in degradation kinetics is primarily due to the fixed generation rate of ROS from PMS activation under constant catalyst and oxidant dosages [56,57,58,59].

The degradation kinetics at different initial TC concentrations were analyzed using the pseudo-first-order kinetic model. The linear fits of −ln(C_0_/C_t_) versus reaction time (R^2^ > 0.97) in Figure 3f confirm pseudo-first-order kinetics for TC degradation at all concentrations tested. Seen in Table S5, the k_obs_ values exhibited a decreasing trend with increasing initial TC concentration. The rate constant decreased from 0.3042 min^−1^ at 10 mg/L TC to 0.1107 min^−1^ at 50 mg/L TC, representing a 2.75-fold reduction as the initial TC concentration increased. The decreasing k_obs_ with increasing initial TC concentration is characteristic of heterogeneous catalytic systems where the generation rate of ROS remains relatively constant under fixed catalyst and oxidant dosages, while the target pollutant concentration varies. Moreover, the double-logarithmic plot of ln(k_obs_) versus ln(C_0_) exhibited excellent linearity (R^2^ = 0.9966) in Figure S7. The slope of the regression line yielded a reaction order (γ) of –0.6146 with respect to initial TC concentration. The negative value indicates that increasing the initial pollutant concentration inhibits the apparent degradation rate, which is consistent with the concept of constant ROS generation rate in the system. The negative reaction order (γ = –0.6146) observed for the initial TC concentration provides important insights into the reaction mechanism of the Fe_3_O_4_@MS/PMS system. This negative-order dependence can be rationalized by considering the competition between TC molecules and ROS generated from PMS activation. The negative-order kinetics are characteristic of heterogeneous advanced oxidation processes where the rate-limiting step is the generation of ROS rather than the reaction between ROS and the target pollutant. The initial TC concentration range of 10–50 mg/L was selected based on (i) its widespread use in TC degradation studies, enabling direct comparison with the literature, (ii) the requirements for reliable kinetic analysis and intermediate identification by LC-MS, and (iii) its relevance to industrial and hospital wastewater streams where antibiotics are present at elevated concentrations. While we acknowledge that TC is detected at lower concentrations (μg/L to ng/L) in natural water bodies, the mechanistic insights and kinetic model developed in this study provide a fundamental understanding that is applicable across a range of concentrations. Future studies will address the degradation performance at environmentally relevant low concentrations using more sensitive analytical techniques.

#### 3.3.4. Effect of Temperature and Thermodynamic Parameters Determination

Temperature is a critical operational parameter that significantly influences the degradation kinetics of TC in the Fe_3_O_4_@MS/PMS system, as it affects both the rate of ROS generation and the molecular diffusion dynamics. To systematically investigate the effect of temperature on TC degradation kinetics, batch experiments were conducted at five different temperatures (20, 25, 30 and 35 °C), while maintaining other operational parameters constant (TC concentration = 50 mg/L, catalyst dosage = 0.5 g/L, PMS concentration = 1.0 mmol/L, initial pH = 6.8). The degradation profiles, kinetic fitting and calculated kinetic parameters are presented in Figure 3g,h and Table S6.

As illustrated in Figure 3g, the degradation efficiency of TC exhibited a pronounced positive correlation with reaction temperature. As the temperature was increased from 20 °C to 35 °C, the TC removal efficiency after 60 min gradually improved from 95.68% to 99.78%. Several factors account for this temperature-dependent enhancement. Higher temperatures boost molecular kinetic energy, promoting more frequent and energetic collisions between PMS and surface Fe^2+^/Fe^3+^ sites on Fe_3_O_4_@MS, which in turn accelerates the production of SO_4_^•−^ and •OH. Secondly, elevated temperatures accelerate the mass transfer of TC from the bulk liquid to the catalyst surface, facilitating more efficient contact between the target pollutant and the generated ROS. Thirdly, elevated temperatures promote the decomposition of PMS and the regeneration of Fe^2+^ from Fe^3+^, sustaining the catalytic cycle [54,60,61,62]. Additionally, the pseudo-first-order model was applied to analyze the degradation kinetics across different temperatures. As seen in Figure 3h and Table S6, the linear relationships between −ln(C_t_/C_o_) and reaction time (R^2^ > 0.95) confirmed the applicability of this model across all investigated temperatures. The k_obs_ values exhibited a substantial increase with rising temperature. At 20 °C, the rate constant was 0.08540 min^−1^. The k_obs_ values rose progressively with temperature: 0.1107 min^−1^ at 25 °C, 0.1307 min^−1^ at 30 °C, and 0.1469 min^−1^ at 35 °C, representing a 1.72-fold increase from 20 °C to 35 °C. This temperature-dependent rise in k_obs_ confirms that the degradation reaction is thermally activated.

To determine the activation energy (E_a_) of the TC degradation process, the temperature-dependent rate constants were analyzed using the Arrhenius equation (Equation (2)):

(2) $$\mathrm{lnk}=\mathrm{lnA}-\frac{\mathrm{Ea}}{RT}$$

where k represents the rate constant (min^−1^), A the pre-exponential factor (min^−1^), E_a_ the activation energy (kJ/mol), R the universal gas constant (8.314 J/mol·K), and *T* the absolute temperature (K). Figure S8 shows the Arrhenius plot of ln k vs. 1/*T*, giving a highly linear fit (R^2^ = 0.9750). From the regression slope and intercept, the activation energy Ea and pre-exponential factor A were determined as 27.04 kJ/mol and 5822.01 min^−1^, respectively, a moderate Ea indicating that Fe_3_O_4_@MS effectively reduces the energy barrier for PMS activation, thus allowing efficient TC degradation under mild temperatures. This characteristic is advantageous for practical wastewater treatment applications, as it implies that satisfactory degradation performance can be achieved without the need for excessive energy input. The activation energy of 27.04 kJ/mol suggests that the rate-determining step is a surface-mediated chemical reaction rather than a diffusion-controlled process. Typically, diffusion-controlled reactions have activation energies below 20 kJ/mol, while chemically controlled reactions exhibit Ea in the range of 20–40 kJ/mol of Fe-based catalysts for activating PMS [63,64,65]. The value obtained in this study (27.04 kJ/mol) therefore indicates that the overall degradation rate is limited by the surface chemical reactions, specifically the electron transfer from Fe^2+^ to adsorbed PMS molecules and the subsequent generation of ROS.

To elucidate the thermodynamic aspects of the degradation process, the Eyring–Polanyi equation (Equation (3)) was further used to determine the enthalpy (Δ*H*) and entropy (Δ*S*) of activation for the degradation process.

(3) $$\ln\left(\frac{k}{T}\right)=\ln(\frac{k_{B}}{h})+\frac{\Delta S}{R}-\frac{\Delta H}{RT}$$

where k_B_, h, and *T* denote the Boltzmann constant (1.3806 × 10^−23^ J/K), Planck constant (6.626 × 10^−34^ J·s), and absolute temperature (K), respectively. The ln(k/*T*) vs. 1/*T* plot (Figure S9) showed good linearity (R^2^ = 0.9696), from whose slope and intercept the activation enthalpy (Δ*H*) and entropy (Δ*S*) were obtained as 25.54 kJ/mol and −195.99 J/mol·K.

The positive Δ*H* (25.54 kJ/mol) indicates that the degradation process is endothermic, consistent with the observed increase in reaction rate with temperature. The negative value of Δ*S* (–195.99 J/mol·K) indicates that the formation of the activated complex is accompanied by a decrease in entropy, suggesting a more ordered transition state compared to the reactants. This negative entropy of activation is characteristic of bimolecular reactions or reactions involving adsorption steps, where the activated complex becomes more constrained due to the formation of surface-bound intermediates. The negative ΔS (–195.99 J/mol·K) provides further mechanistic insights. This negative ΔS implies a more ordered activated complex relative to the reactants, which is consistent with a mechanism involving the adsorption of PMS onto the Fe_3_O_4_@MS surface prior to the rate-determining step [66,67].

### 3.4. Effect of pH and Coexisting Anions on TC Degradation

**Figure 4:**
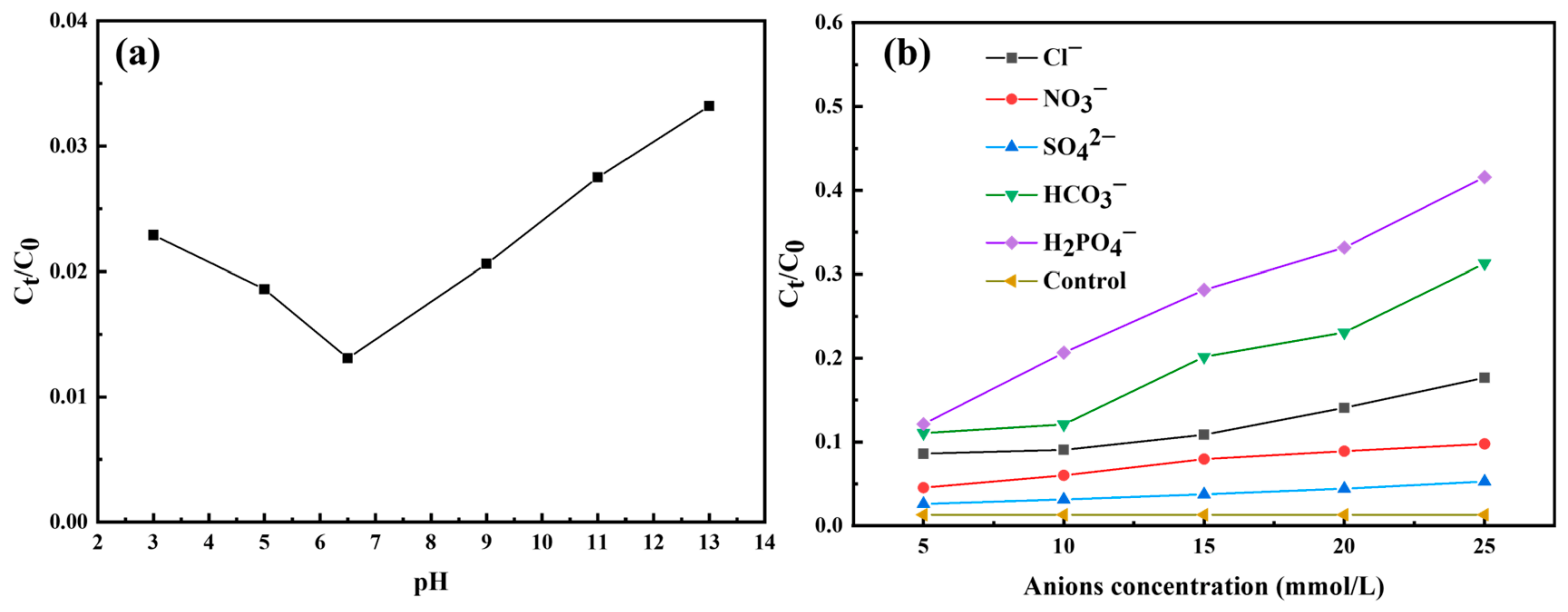
(**a**) TC degradation under different initial pH (Experimental conditions: TC concentration = 50 mg/L, PMS concentration = 1.0 mmol/L, catalyst dosage = 0.5 g/L, reaction time = 60 min, temperature = 25 °C); (**b**) TC degradation under different anions concentrations (Experimental conditions: TC concentration = 50 mg/L, PMS concentration = 1.0 mmol/L, catalyst dosage = 0.5 g/L, reaction time = 60 min, initial pH = 6.8, temperature = 25 °C).

The practical application of heterogeneous advanced oxidation processes is often influenced by water matrix components, including solution pH and coexisting inorganic anions. To evaluate the applicability of the Fe_3_O_4_@MS/PMS system under realistic conditions, systematic investigations were performed to evaluate the influence of initial pH and common coexisting anions (Cl^−^, HCO_3_^−^, NO_3_^−^, H_2_PO_4_^−^ and SO_4_^2−^) on TC degradation performance. The results are presented in Figure 4a,b, illustrating the degradation profiles under different pH conditions and the influence of various anions, respectively.

As illustrated in Figure 4a, when the initial pH was adjusted to 3.0, 5.0, and 6.5, the degradation efficiencies were 97.71%, 98.14%, and 98.69%, respectively, indicating that the system maintained high catalytic activity under both acidic and near-neutral conditions. TC is an amphoteric molecule with three ionizable functional groups (pK_a1_ = 3.3, pK_a2_ = 7.7, pK_a3_ = 9.7), allowing it to exist in up to four different protonation states (H_3_TC^+^, H_2_TC, HTC^−^, and TC^2−^) depending on the solution pH. Under acidic to near-neutral conditions (pH 3.0–6.5), TC exists predominantly as a cationic or zwitterionic species, which can enhance electrostatic attraction to the negatively charged catalyst surface. This favorable electrostatic interaction promotes the adsorption of TC molecules onto the catalyst surface, facilitating subsequent oxidation by surface-generated ROS. The Fe_3_O_4_@MS/PMS system exhibits an optimal pH range of 3.0–9.0, although even under strongly alkaline conditions (pH 13.0), the degradation efficiency is still as high as 96.68% after 60 min. Namely, the Fe_3_O_4_@MS/PMS system exhibited excellent TC degradation performance across a broad pH range of 3.0–13.0. The decrease in degradation efficiency under highly alkaline conditions can be attributed to the following reasons. Firstly, under highly alkaline conditions, PMS predominantly exists as SO_5_^2−^, which is less reactive and more difficult to activate via surface-bound iron species compared to HSO_5_^−^. And the deprotonated SO_5_^2−^ species undergoes intrinsic decomposition and this decomposition reduces the effective PMS concentration available for activation. Secondly, at high pH, hydroxide ions (OH^−^) compete with the target pollutant for sulfate radicals, leading to the conversion of SO_4_^•−^ to hydroxyl radicals (•OH) (SO_4_^•−^ + OH^−^ → SO_4_^2−^ + •OH). This conversion reduces the overall oxidation capacity because •OH has a shorter half-life than SO_4_^•−^. Additionally, both the catalyst surface and TC molecules become negatively charged, resulting in electrostatic repulsion that hinders TC adsorption and reduces degradation efficiency under highly alkaline conditions. The excellent performance across a broad pH range (3.0–13.0) indicates that the system is suitable for treating TC-contaminated wastewater without the need for extensive pH adjustment, which is advantageous for reducing operational costs and simplifying treatment processes.

Meanwhile, as presented in Figure 4b, the addition of Cl^−^ exhibited a concentration-dependent inhibitory effect on TC degradation. At a Cl^−^ concentration of 5 mmol/L, the TC degradation efficiency after 60 min decreased from 98.69% (control without anions) to 91.40%. As the Cl^−^ concentration increased to 10, 15, 20 and 25 mmol/L, the degradation efficiencies further decreased to 90.94%, 89.14%, 85.92%, and 82.33%, respectively. The inhibitory effect of Cl^−^ can be attributed to the scavenging of sulfate radicals (SO_4_^•−^) and hydroxyl radicals (•OH) by chloride ions, as described by Equations (4) and (5). NO_3_^−^ exhibited relatively weak inhibitory effects on TC degradation compared to Cl^−^ and HCO_3_^−^. At NO_3_^−^ concentrations of 5, 10, 15, 20, and 25 mmol/L, the TC degradation efficiencies after 60 min were 95.45%, 93.99%, 92.05%, 91.11%, and 90.23%, respectively, compared to 98.69% in the control. The weak inhibitory effect of NO_3_^−^ is attributed to its low reactivity with SO_4_^•−^ and •OH. The reaction rate constants for NO_3_^−^ with SO_4_^•−^ and •OH are significantly lower than those for Cl^−^ and HCO_3_^−^. As for SO_4_^2−^, SO_4_^2−^ exhibited the weakest inhibitory effect among all tested anions. At SO_4_^2−^ concentrations of 5, 10, 15, 20 and 25 mmol/L, the TC degradation efficiencies after 60 min were 9740%, 96.86%, 96.25%, 95.58%, and 94.73%, respectively. The minimal interference of SO_4_^2−^ can be explained by its very low reactivity with both SO_4_^•−^ and •OH. The reaction rate constants for SO_4_^2−^ with these radicals are negligible, and sulfate ions do not participate in significant radical scavenging reactions under typical conditions. H_2_PO_4_^−^ exhibited a strongest inhibitory effect on TC degradation among all tested anions, with pronounced concentration dependence [21,68,69]. At H_2_PO_4_^−^ concentrations of 5, 10, 15, 20, and 25 mmol/L, the TC degradation efficiencies after 60 min were 87.87%, 79.34%, 71.86%, 66.85%, and 58.43%, respectively. The strong inhibition by H_2_PO_4_^−^ can be attributed to multiple mechanisms. First, H_2_PO_4_^−^ is an effective radical scavenger that reacts with SO_4_^•−^ and •OH, as described by Equations (6) and (7). Second, H_2_PO_4_^−^ has a strong complexation affinity for iron species. The formation of Fe^3+ –^–phosphate complexes can significantly reduce the availability of active Fe^2+^/Fe^3+^ sites on the catalyst surface, inhibiting the redox cycling that is essential for PMS activation (see Equation (8)). HCO_3_^−^ exhibited a strong inhibitory effect on TC degradation, comparable to H_2_PO_4_^−^. At HCO_3_^−^ concentrations of 5, 10, 15, 20, and 25 mmol/L, the TC degradation efficiencies after 60 min were 88.94%, 87.91%, 79.84%, 76.92%, and 68.73%, respectively. The strong inhibition by HCO_3_^−^ is primarily due to its high reactivity with both SO_4_^•−^ and •OH (see Equations (9) and (10)). Given above, the inhibitory effects followed the order: H_2_PO_4_^−^ > HCO_3_^−^ > Cl^−^ > NO_3_^−^ > SO_4_^2−^ at all investigated concentrations. The concentration dependence was most pronounced for H_2_PO_4_^−^ and HCO_3_^−^, while SO_4_^2−^ and NO_3_^−^ exhibited relatively weak concentration-dependent inhibition.

(4) $${\mathrm{SO}}_{4}^{•-}+{\mathrm{Cl}}^{-}\to {\mathrm{SO}}_{4}^{2-}+{\mathrm{Cl}}^{•}$$

(5) $$•\mathrm{OH}+{\mathrm{Cl}}^{-}\to {\mathrm{ClOH}}^{•-}$$

(6) $${\mathrm{SO}}_{4}^{•-}+H_{2}{\mathrm{PO}}_{4}^{-}\to {\mathrm{SO}}_{4}^{2-}+H_{2}{\mathrm{PO}}_{4}^{•}$$

(7) $$•\mathrm{OH}+H_{2}{\mathrm{PO}}_{4}^{-}\to {\mathrm{OH}}^{-}+H_{2}{\mathrm{PO}}_{4}^{•}$$

(8) $${\mathrm{Fe}}^{3+}+H_{2}{\mathrm{PO}}_{4}^{-}\to {{[\mathrm{Fe}(H}_{2}{\mathrm{PO}}_{4})]}^{2+}$$

(9) $${\mathrm{SO}}_{4}^{•-}+{\mathrm{HCO}}_{3}^{-}\to {\mathrm{SO}}_{4}^{2-}+{\mathrm{CO}}_{3}^{•}+H^{+}$$

(10) $$•\mathrm{OH}+{\mathrm{HCO}}_{3}^{-}\to {\mathrm{CO}}_{3}^{•-}+H_{2}O$$

### 3.5. Reusability and Stability of Fe_3_O_4_@MS

The reusability and stability of a heterogeneous catalyst are critical factors for evaluating its practical applicability and economic feasibility in wastewater treatment. To assess the long-term performance of the Fe_3_O_4_@MS catalyst synthesized from CSS, consecutive degradation cycles were conducted under optimized conditions (TC = 50 mg/L, catalyst = 0.5 g/L, PMS = 1.0 mmol/L, reaction time = 60 min, initial pH = 6.8, temperature = 25 °C). The results are presented in Figure S10, illustrating the degradation efficiency across five consecutive cycles and iron leaching concentrations.

As shown in Figure S10a, the Fe_3_O_4_@MS catalyst maintained excellent catalytic activity over five consecutive degradation cycles. In the first cycle, the TC degradation efficiency reached 96.47% after 60 min of reaction. In the second, third, fourth, and fifth cycles, the degradation efficiencies were 94.54%, 92.64%, 91.74%, and 90.55%, respectively. The slight decrease in degradation efficiency over successive cycles can be attributed to some factors, including the accumulation of adsorbed intermediates on the catalyst surface, minor loss of active iron species through leaching, and potential partial oxidation or structural changes in the Fe_3_O_4_ nanoparticles. Catalyst stability was further assessed by quantifying leached iron ions in the post-reaction solution after each cycle. As shown in Figure S10b, the iron leaching concentration for fresh Fe_3_O_4_@MS was 0.21 mg/L. While in the first, second, third, fourth, and fifth cycles, the iron leaching concentrations were both lower than 0.20 mg/L. The low and gradually decreasing iron leaching values indicate that the Fe_3_O_4_ nanoparticles are firmly anchored within the mesoporous silica matrix, preventing significant loss of active species during the reaction. The iron leaching concentrations are well below the discharge limits for iron in wastewater (typically < 2 mg/L), confirming the environmental compatibility of the catalyst. To verify the mineralization products, we conducted IC analysis for NO_3_^−^ of the reaction solution under optimal degradation conditions. Since the complete mineralization of 50 mg/L TC would theoretically yield 13.95 mg/L NO_3_^−^ (if all N was converted to NO_3_^−^). The actual IC-detected value was 6.14 mg/L NO_3_^−^ and mass balance analysis confirms that the nitrogen recovery reaches 44.01% (6.14/13.95). These results indicate that the nitrogen atoms in the TC molecule (containing dimethylamino and amide groups) were partially mineralized to nitrate during the oxidation process, consistent with the proposed degradation pathway involving ring-opening and demethylation reactions. The washing step would remove any nitrate or other inorganic nitrogen species from the catalyst surface, as these are highly water-soluble and do not adsorb strongly to the Fe_3_O_4_@MS surface. Moreover, this concentration is well below the regulatory limits for drinking water (10 mg N/L, GB 5749-2022; 50 mg NO_3_^−^/L, WHO guideline), surface water (10 mg N/L for Class I–III, GB 3838-2002), groundwater (20 mg N/L for Class III, GB/T 14848-2017), and wastewater discharge (15 mg N/L for primary discharge, GB 8978-1996). Therefore, the residual nitrate generated during the Fe_3_O_4_@MS/PMS treatment process does not pose a significant secondary contamination risk.

After separation and washing, the residual TC concentration in the supernatant was determined by UV–vis spectrophotometry at 357 nm. After 5 successive cycles, the degradation efficiency decreased to 90.55% (5th cycle), which means that approximately 9.45% of the initial TC (equivalent to 4.73 mg/L from an initial concentration of 50 mg/L) remained undegraded. And the residual TC concentration in the effluent was 1.38 mg/L, indicating that 2.76% of the initial TC remained undegraded in the reaction solution. The rest of undegraded TC (approximately 6.69%) was mainly adsorbed on the surface of Fe_3_O_4_@MS. The catalyst washing step (with deionized water and ethanol) is designed to remove loosely bound species from the catalyst surface before the next cycle. Therefore, this residual TC, along with adsorbed intermediates on the catalyst surface, can been effectively removed during the washing/regeneration step. And the desorbed TC amount was quantified as 6.59 mg/g-catalyst, indicating that 98.56% of the TC adsorbed on the Fe_3_O_4_@MS surface has been effectively eluted.

### 3.6. Contribution Rate of ROS

**Figure 5:**
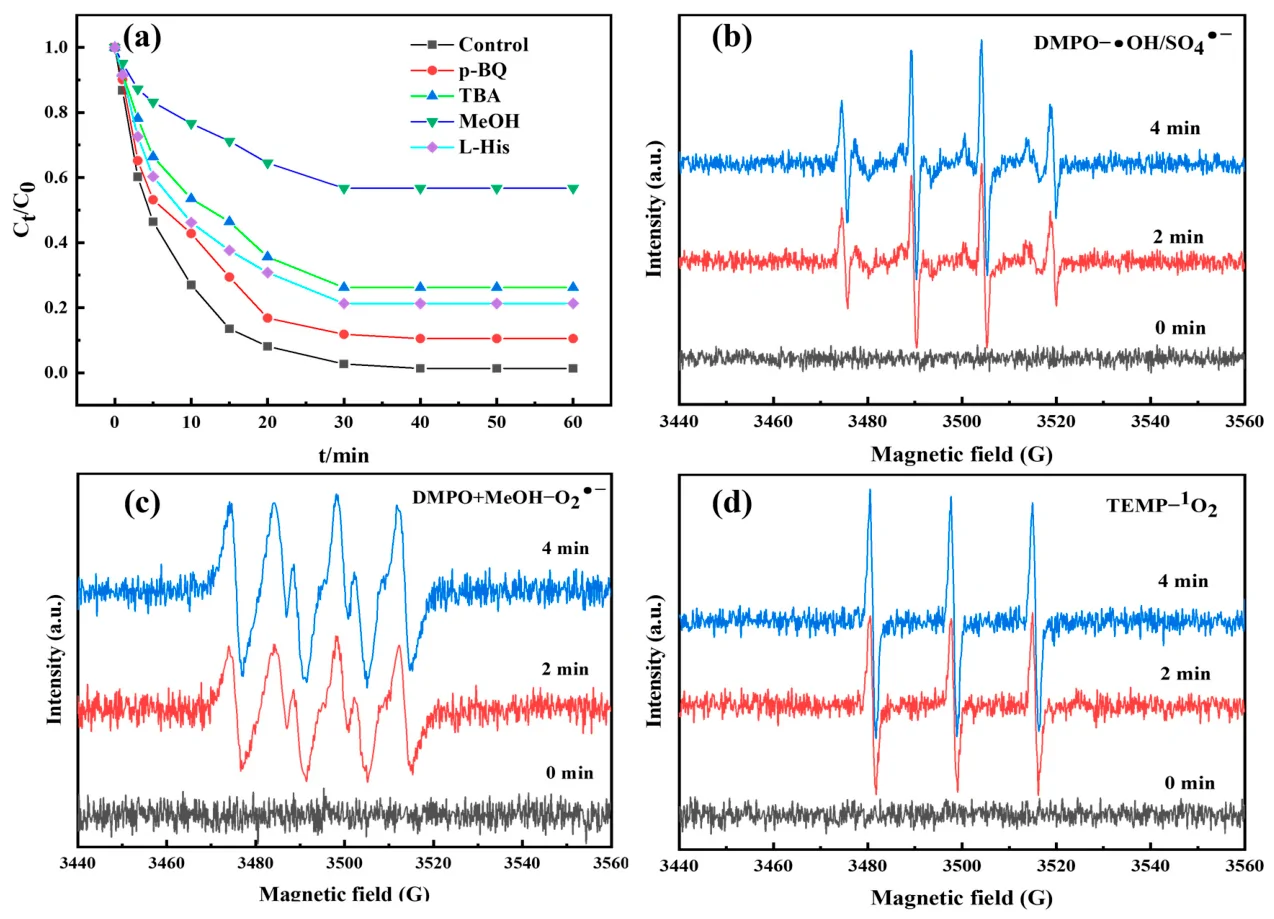
(**a**) Effects of different radical scavengers on TC degradation in the Fe_3_O_4_@MS/PMS system: methanol (MeOH, 100 mmol/L, scavenging both SO_4_^•−^ and •OH), tert-butanol (TBA, 100 mmol/L, preferentially scavenging •OH), p-benzoquinone (p-BQ, 10 mmol/L, scavenging O_2_^•−^) and L-histidine (L-his, 10 mmol/L, scavenging ^1^O_2_). Experimental conditions: TC concentration = 50 mg/L, PMS concentration = 1.0 mmol/L, catalyst dosage = 0.5 g/L, initial pH = 6.8, temperature = 25 °C. (**b**–**d**) EPR spectra of the Fe_3_O_4_@MS/PMS system recorded after 2 and 4 min of reaction: (**b**) DMPO–•OH together with the DMPO–SO_4_^•−^ adduct signals; (**c**) DMPO–O_2_^•−^ adduct signals; (**d**) TEMP–^1^O_2_ adduct. Abbreviations: EPR, electron paramagnetic resonance; DMPO, 5,5-dimethyl-1-pyrroline N-oxide; TEMP, 2,2,6,6-tetramethylpiperidine.

To identify the primary ROS driving TC degradation within the Fe_3_O_4_@MS/PMS system and to quantitatively evaluate their respective contributions, quenching tests were performed with selected scavengers. The experiments were performed under optimized conditions (TC = 50 mg/L, catalyst = 0.5 g/L, PMS = 1.0 mmol/L, initial pH = 6.8, temperature = 25 °C) with the addition of various scavengers prior to the initiation of the reaction. The degradation profiles with different scavengers’ results are presented in Figure 5. And the corresponding pseudo-first-order fitting under different scavengers and the calculated contribution rates of each ROS were listed in Figure S11 and Table S7. As illustrated in Figure 5a and Figure S11, and Table S7, the addition of different scavengers resulted in varying degrees of inhibition on TC degradation, providing insights into the roles of different ROS. In the control experiment without any scavenger, TC degradation reached 98.69% after 60 min, with a corresponding k_obs_ of 0.1104 min^−1^. Upon the addition of MeOH (100 mmol/L), which scavenges both SO_4_^•−^ and •OH, TC degradation efficiency decreased significantly to 43.33% after 60 min, with the k_obs_ value dropping to 0.0181 min^−1^. This represents an 83.61% reduction in the rate constant, indicating that SO_4_^•−^ and •OH collectively play a dominant role in TC degradation. When TBA (100 mmol/L) was added as a selective •OH scavenger, TC degradation efficiency decreased to 73.80% after 60 min, with the k_obs_ value decreasing to 0.0440 min^−1^. This represents a 60.14% reduction compared to the control, suggesting that •OH contributes significantly to the overall degradation. The addition of p-BQ (10 mmol/L) as an O_2_^•−^ scavenger resulted in a moderate inhibition, with TC degradation efficiency decreasing to 89.50% after 60 min and the k_obs_ value decreasing to 0.0720 min^−1^, representing a 34.78% reduction. This indicates that O_2_^•−^ also participates in TC degradation, albeit to a lesser extent compared to SO_4_^•−^ and •OH. When L-His (10 mmol/L) was added as a ^1^O_2_ scavenger, TC degradation efficiency decreased to 78.72% after 60 min, with the k_obs_ value decreasing to 0.0506 min^−1^, representing a 54.17% reduction. This suggests that ^1^O_2_, generated through non-radical pathways, contributes substantially to TC degradation.

EPR spectroscopy was also employed to directly identify and characterize the ROS generated in the Fe_3_O_4_@MS/PMS system. The EPR spectra of DMPO spin adduct are shown in Figure 5b. The presence of both DMPO-OH and DMPO-SO_4_ signals confirms the simultaneous generation of •OH and SO_4_^•−^ in the Fe_3_O_4_@MS/PMS system. The significantly higher intensity of the DMPO-OH signal compared to the DMPO-SO_4_ signal indicates that •OH is the dominant radical species under the experimental conditions. This observation is consistent with the radical quenching results, which showed a higher contribution rate for •OH (60.14%) compared to SO_4_^•−^ (23.47%). The signal intensity of O_2_^•−^ increased progressively with reaction time, indicating the continuous generation of O_2_^•−^ throughout the degradation process (see Figure 5c). The spectrum exhibited a characteristic three-line signal with equal intensity (1:1:1), which is typical for the TEMP radical formed by the reaction of TEMP with ^1^O_2_. The strong signal intensity indicates that ^1^O_2_ is generated abundantly in the Fe_3_O_4_@MS/PMS system (see Figure 5d). The significant contribution of ^1^O_2_ (54.17% from radical quenching experiments) is corroborated by the strong EPR signal observed. The EPR results provide direct experimental evidence for the generation of multiple ROS in the Fe_3_O_4_@MS/PMS system, including •OH, SO_4_^•−^, O_2_^•−^, and ^1^O_2_. The dominance of •OH, as indicated by the strong DMPO-OH signal, explains the high degradation efficiency of TC, as •OH is a non-selective oxidant capable of attacking various functional groups in the TC molecule. The presence of ^1^O_2_, as confirmed by the strong TEMPO signal, indicates that non-radical pathways play an important role in the degradation process. The detection of O_2_^•−^ confirms its role as an intermediate linking radical and non-radical pathways, participating in the generation of both •OH and ^1^O_2_.

Based on the comprehensive analysis of radical quenching experiments and EPR spectroscopy of the Fe_3_O_4_@MS catalyst, the radical transformation pathways in the Fe_3_O_4_@MS/PMS system are proposed. The generation, interconversion, and synergistic interactions among various ROS, including SO_4_^•−^, •OH, O_2_^•−^, and ^1^O_2_, are elucidated. The catalytic activation of PMS by the Fe_3_O_4_@MS catalyst initiates the cascade of radical generation. The Fe_3_O_4_ nanoparticles uniformly dispersed within the mesoporous silica matrix provide abundant Fe^2+^ active sites on the catalyst surface. When PMS (HSO_5_^−^) approaches the catalyst surface, electron transfer occurs from Fe^2+^ to the O–O bond of HSO_5_^−^, leading to the generation of SO_4_^•−^ and •OH through two distinct pathways via Equations (11) and (12).

(11) $${\mathrm{Fe}}^{2+}+{\mathrm{HSO}}_{5}^{-}\to {\mathrm{Fe}}^{3+}+{\mathrm{SO}}_{4}^{•-}+{\mathrm{OH}}^{-}$$

(12) $${\mathrm{Fe}}^{2+}+{\mathrm{HSO}}_{5}^{-}\to {\mathrm{Fe}}^{3+}+{\mathrm{SO}}_{4}^{2-}+•\mathrm{OH}$$

For sustained catalytic activity, the regeneration of Fe^2+^ from Fe^3+^ is essential. The Fe_3_O_4_@MS catalyst facilitates this redox cycling through multiple mechanisms. The Fe^3+^ generated from Equations (11) and (12) can be reduced back to Fe^2+^ by reaction with excess HSO_5_^−^ (Equation (13)). Additionally, the inherent mixed-valence structure of Fe_3_O_4_ (containing both Fe^2+^ and Fe^3+^ in octahedral sites) facilitates electron transfer between iron centers, promoting the regeneration of surface Fe^2+^ sites (Equation (14)).

(13) $${\mathrm{Fe}}^{3+}+{\mathrm{HSO}}_{5}^{-}\to {\mathrm{Fe}}^{2+}+{\mathrm{SO}}_{5}^{•-}+H^{+}$$

(14) $${\mathrm{Fe}}^{3+}+{\mathrm{Fe}}^{2+}⇌{\mathrm{Fe}}^{3+}+{\mathrm{Fe}}^{2+}$$

The interconversion between SO_4_^•−^ and •OH plays a crucial role in determining the dominant radical species in the system. The contribution rate analysis revealed that •OH is the dominant radical species (60.14%), while SO_4_^•−^ contributes 23.47%. This predominance of •OH is attributed to the efficient conversion of SO_4_^•−^ to •OH through reactions with water and hydroxide ions via Equations (15) and (16).

(15) $${\mathrm{SO}}_{4}^{•-}+H_{2}O\to •\mathrm{OH}+{\mathrm{SO}}_{4}^{2-}+H^{+}$$

(16) $${\mathrm{SO}}_{4}^{•-}+{\mathrm{OH}}^{-}\to •\mathrm{OH}+{\mathrm{SO}}_{4}^{2-}$$

O_2_^•−^ contribute approximately 34.78% to TC degradation in the Fe_3_O_4_@MS/PMS system. The generation of O_2_^•−^ occurs through multiple pathways: (1) Dissolved oxygen in the aqueous solution can react with surface Fe^2+^ sites to generate O_2_^•−^ (Equation (17)); (2) the SO_5_^•−^ radical can further undergo decomposition to generate O_2_^•−^ (Equation (18)).

(17) $${\mathrm{Fe}}^{2+}+O_{2}\to {\mathrm{Fe}}^{3+}+O_{2}^{•-}$$

(18) $${\mathrm{SO}}_{5}^{•-}\to {\mathrm{SO}}_{4}^{2-}+O_{2}^{•-}+H^{+}$$

The significant contribution of ^1^O_2_ (54.17%) to TC degradation indicates the importance of non-radical pathways in the Fe_3_O_4_@MS/PMS system. The generation of ^1^O_2_ proceeds through three distinct mechanisms: (a) PMS can undergo self-decomposition in the presence of the catalyst surface, generating ^1^O_2_ through a non-radical pathway (Equation (19)); (b) superoxide radicals can recombine to generate ^1^O_2_ through a bimolecular reaction (Equation (20)); (c) the reaction between superoxide radicals and hydroxyl radicals provides another route for ^1^O_2_ generation (Equation (21)).

(19) HSO5−+SO52−→HSO4−+SO42−+O21

(20) 2O2•+2H+→H2O2+O21

(21) O2•−+•OH→OH−+O21

### 3.7. Surface Chemical Reactions

**Figure 6:**
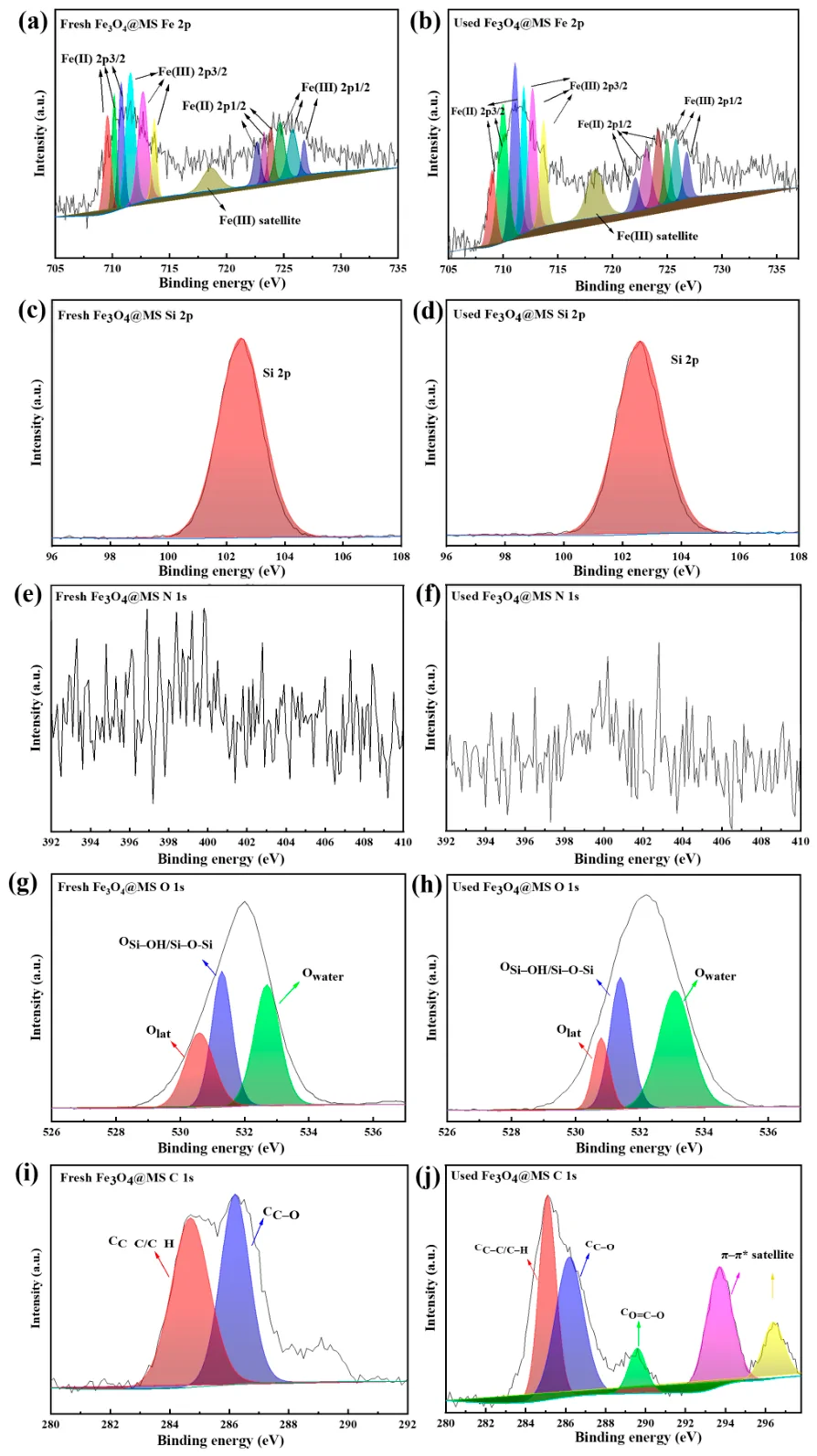
High-resolution XPS spectra of the fresh (left panels (**a**,**c**,**e**,**g**,**i**)) and used (right panels (**b**,**d**,**f**,**h**,**j**)) Fe_3_O_4_@MS catalyst (used = recovered after 5 degradation cycles under the conditions given in Figure S10), compared to identify the surface changes induced by PMS activation and TC degradation: (**a**,**b**) Fe 2p; (**c**,**d**) Si 2p; (**e**,**f**) N 1s; (**g**,**h**) O 1s; (**i**,**j**) C 1s.

High-resolution XPS (Fe 2p, Si 2p, O 1s, N 1s, C 1s) was performed on fresh and used catalysts to probe the interaction of TC and its intermediates with the Fe_3_O_4_@MS surface. The high-resolution Fe 2p spectra of fresh and spent Fe_3_O_4_@MS are shown in Figure 6a,b. The fresh catalyst spectrum exhibited two main peaks at binding energies of 711.2 eV and 724.6 eV, corresponding to Fe 2p_3/2_ and Fe 2p_1/2_, respectively. Peak deconvolution of Fe 2p_3/2_ yielded two components at 710.8 eV and 712.2 eV, assigned to Fe^2+^ and Fe^3+^, respectively, and the calculated Fe^2+^/Fe^3+^ ratio on the fresh catalyst surface was 0.72. After the degradation reaction (used catalyst), the Fe 2p spectrum exhibited a slight shift toward higher binding energy, with the Fe 2p_3/2_ peak centered at 711.5 eV. Deconvolution of the used catalyst Fe 2p_3/2_ peak gave Fe^2+^ and Fe^3+^ components at 711.1 eV and 712.7 eV, respectively. The Fe^2+^/Fe^3+^ ratio dropped to 0.65, demonstrating partial oxidation of surface Fe^2+^ to Fe^3+^ during the repeated PMS activation cycles (Equations (11)–(14)). The slight positive shift in binding energies also suggests changes in the local coordination environment of iron, potentially arising from either surface iron–hydroxyl complexation or sulfate species adsorption from the reaction medium. The Si 2p profiles of fresh and used Fe_3_O_4_@MS (Figure 6c,d) exhibited a single peak at approximately 102.6 eV, characteristic of Si^4+^ in the SiO_4_ tetrahedra of the mesoporous silica framework. Negligible variation in binding energy or change in peak shape was observed after the degradation, demonstrating that the silica support maintained chemically stable under the reaction conditions. The minor loss in peak intensity likely results from organic intermediates depositing on the catalyst surface and partially blocking the Si signal. The N 1s high-resolution spectrum of the fresh and used Fe_3_O_4_@MS catalyst (Figure 6e,f) exhibited no detectable nitrogen signal. This observation is consistent with the synthesis process, which involved no nitrogen-containing precursors. And the TC and its nitrogen-containing degradation intermediates show almost no adsorption onto the Fe_3_O_4_@MS surface. The high-resolution O 1s spectra of fresh and used Fe_3_O_4_@MS are presented in Figure 6g,h. Deconvolution of the fresh catalyst’s O 1s spectrum gave three peaks, with the component at 530.6 eV assigned to lattice oxygen (O^2−^) within the Fe–O bonds of Fe_3_O_4_. The peak at 531.3 eV is attributed to surface hydroxyl groups (Si–OH, Fe–OH) and bridging oxygen atoms in the Si–O–Si network of the mesoporous silica support. The peak at 532.7 eV is assigned to adsorbed water molecules (H_2_O) on the catalyst surface. After the degradation reaction, the used catalyst O 1s spectrum showed significant changes in the relative intensities of these components. The lattice oxygen peak (530.6 eV) decreased from 26.91% to 15.02% in intensity relative to the fresh catalyst, consistent with the partial oxidation of Fe_3_O_4_ and possible surface restructuring. The surface hydroxyl peak (531.3 eV) increased substantially, from 34.56% of total O 1s area in the fresh catalyst to 35.49% in the used catalyst. This increase is attributed to the adsorption of TC degradation intermediates and the formation of additional surface hydroxyl groups during the catalytic reaction. The C 1s spectrum of the fresh Fe_3_O_4_@MS catalyst (Figure 6i,j) exhibited a dominant peak at 284.8 eV, characteristic of adventitious carbon (C–C/C–H) commonly present on air-exposed surfaces. This peak arises from atmospheric contamination and is typically unavoidable in XPS analysis. A minor peak at approximately 286.2 eV was also observed, corresponding to carbon atoms bonded to oxygen (C–O), likely originating from residual organic species from the synthesis process. The absence of significant peaks at higher binding energies (288–290 eV) indicated that the fresh catalyst surface was relatively clean, with minimal oxygen-containing functional groups. After five degradation cycles, deconvolution of the used Fe_3_O_4_@MS C 1s spectrum revealed four distinct components, providing detailed information on the types of carbon-containing species present: C–C/C–H at 285.1 eV, C–O at 286.2 eV, O–C=O at 289.6 eV. The presence of oxygenated functional groups, particularly carboxyl groups, provides direct evidence for the adsorption of TC degradation intermediates and supports the proposed ring-opening degradation pathways. Moreover, two additional peaks were observed at approximately 293.7 eV and 296.4 eV. These peaks are attributed to π–π* shake-up satellite transitions associated with the aromatic structures of adsorbed TC degradation intermediates.

### 3.8. DFT Calculations for Active Site

To elucidate the molecular-level reactivity of TC toward different ROS and to identify the preferential attack sites during the degradation process, Materials Studio software (V2019) was employed for density functional theory (DFT) calculations. Fukui indices, which are local reactivity descriptors based on frontier molecular orbital theory, were employed to evaluate the nucleophilic, electrophilic, and radical attack preferences of different atoms and functional groups within the TC molecule. The optimized geometry of TC is presented in Figure S12 and the calculated condensed Fukui indices for the key atoms and functional groups of TC are summarized in Table S8. DFT calculations and Fukui index analysis provided molecular-level insights into the reactivity of TC toward different reactive oxygen species. The phenolic A-ring atoms (C4, C5, C15, O23, O27, N29) exhibited the highest electrophilic Fukui indices (f^−^), identifying them as the primary sites for attack by SO_4_^•−^, •OH, and ^1^O_2_. The B, C-ring atoms (C2, C3, O25, O26) exhibited the highest nucleophilic Fukui indices (f^+^), identifying them as the primary sites for attack by O_2_^•−^. These theoretical predictions are in excellent agreement with the degradation intermediates identified by LC-MS, which showed initial hydroxylation and demethylation followed by deamination and A-ring cleavage (see Section 3.9). The Fukui index analysis provides a theoretical framework for understanding the selective reactivity of TC toward different ROS and validates the proposed degradation pathways.

### 3.9. Degradation Pathway

**Figure 7:**
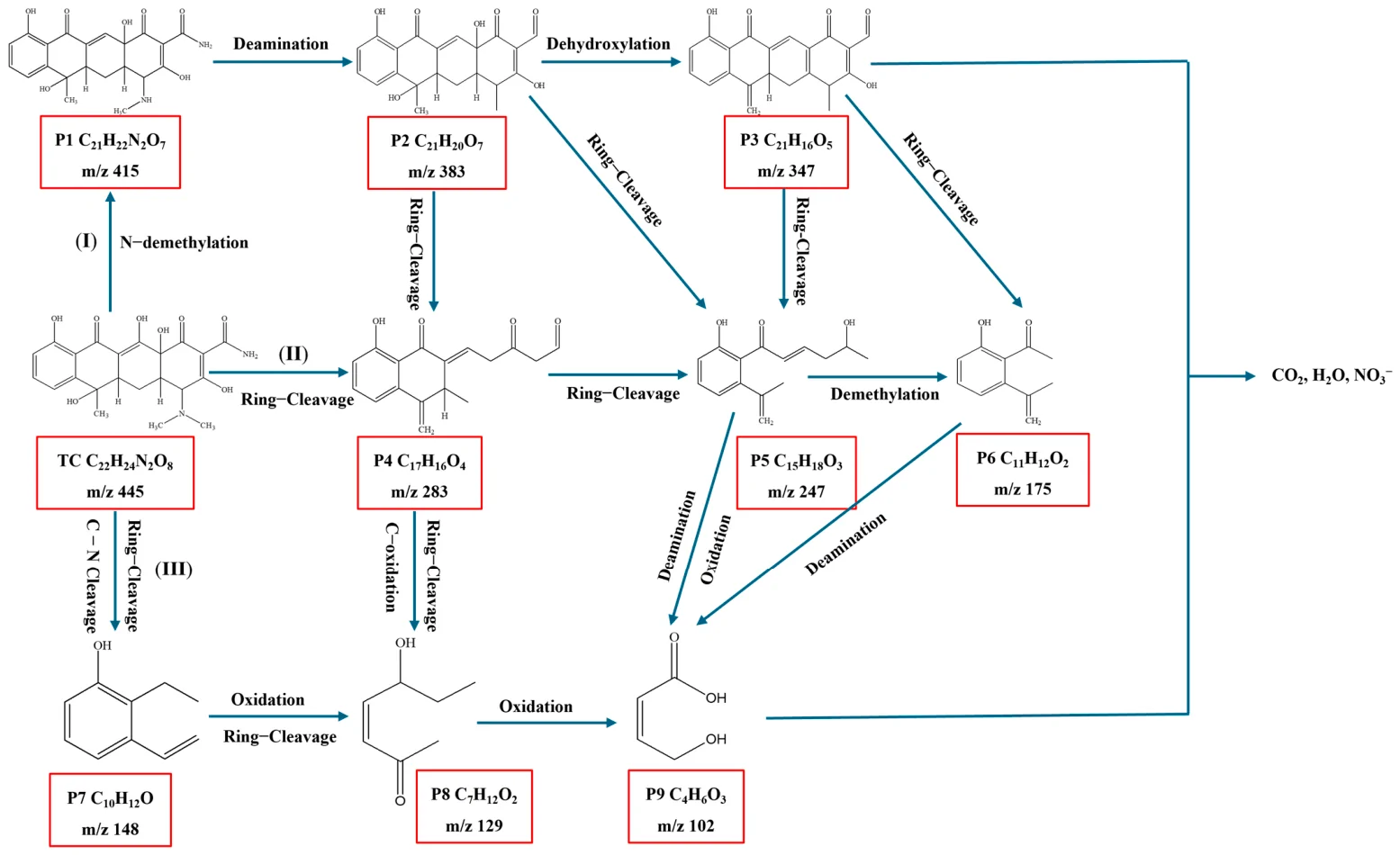
Degradation products and degradation pathway of TC in the Fe_3_O_4_@MS/PMS system.

To elucidate the degradation mechanism of TC in the Fe_3_O_4_@MS/PMS system and to identify the intermediates produced under oxidative conditions, LC-MS analysis was conducted. The identified intermediates and their corresponding molecular ions, *m*/*z* and proposed structures are summarized in Table S9, and the proposed degradation pathways are illustrated in Figure 7. From these intermediates and prior reports, three plausible TC transformation pathways were proposed. The degradation involved N-demethylation, dehydroxylation, deamination, and ring-cleavage. Pathway I involves the continuous removal of functional groups from the tetracyclic structure, which are highly susceptible to radical attack by N-demethylation, dehydroxylation and deamination. The intermediate with *m*/*z* 415.0 indicates the loss of an N-methyl group and a hydroxyl group from the parent TC molecule. Further sequential oxidation and group detachment processes, including deamination and dehydroxylation, lead to the formation of intermediates with *m*/*z* 383.0 and *m*/*z* 347.0. For Pathway II, as the degradation proceeds, the carbon–carbon double bonds and the tetracyclic ring skeleton (Rings A–D) become the primary targets for aggressive ROS attack, leading to ring-opening reactions. The breakdown of the naphthol structure and subsequent ring cleavage produces several lower molecular weight intermediates. Prominent peaks at *m*/*z* 283 and *m*/*z* 247 indicate the successful cleavage of the external rings (A and B rings). Continued oxidative scission of these fragments generates even smaller aromatic and aliphatic intermediates, represented by *m*/*z* 175. In Pathway III of the advanced oxidation process, the ring-cleavage products are further oxidized into small molecular organic acids and fragments to achieve deep oxidation and mineralization. The distinct appearance of a peak at *m*/*z* 148, *m*/*z* 129 and *m*/*z* 102 corresponds to highly degraded, short-chain aliphatic compounds. Ultimately, with sufficient reaction time and PMS activation, these small organic molecules are completely mineralized into CO_2_ and H_2_O.

### 3.10. Toxicity Assessment

**Figure 8:**
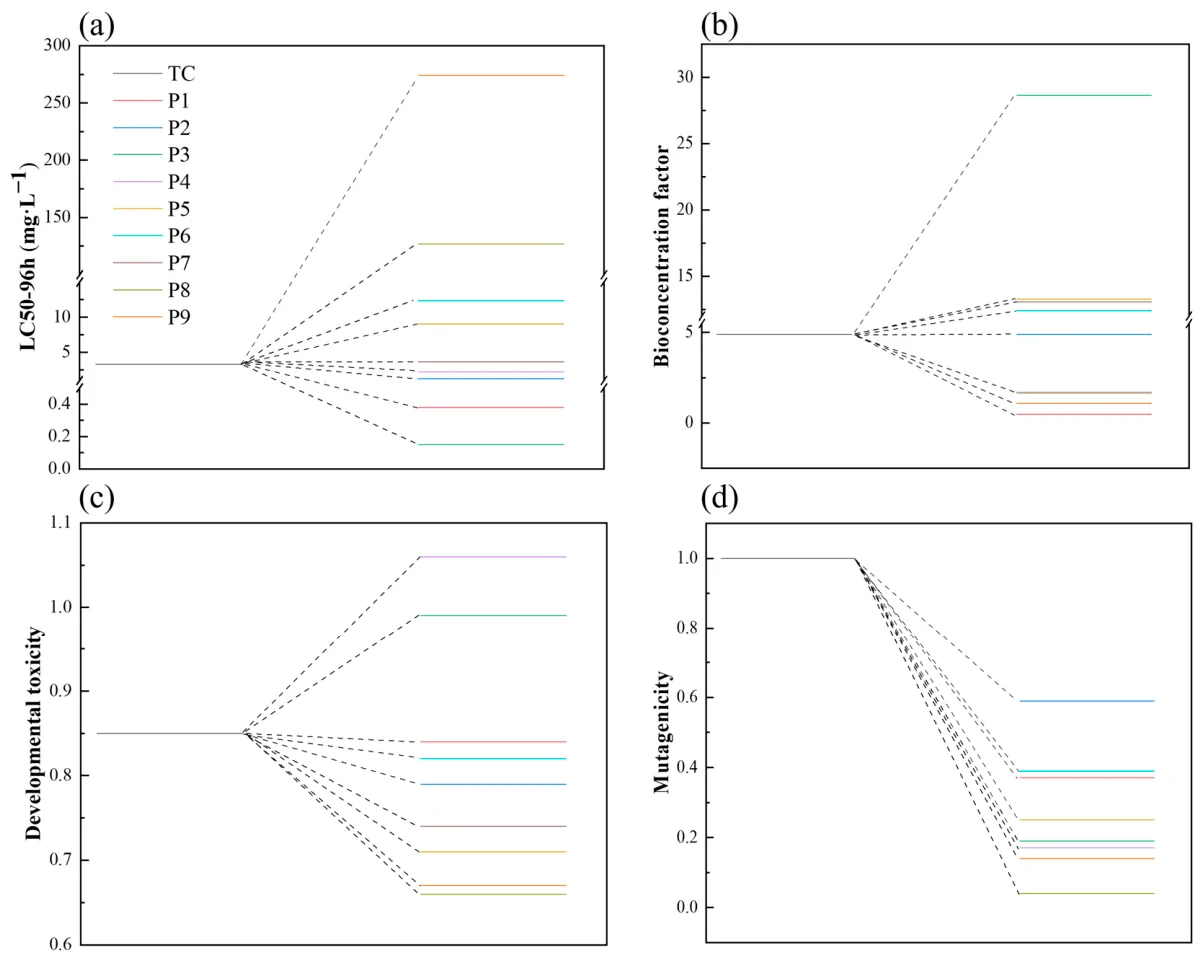
Temporal evolution of the predicted ecotoxicity during the degradation of TC in the Fe_3_O_4_@MS/PMS system estimated with the T.E.S.T. For TC and each intermediate identified at Figure 7, four QSAR-based endpoints were predicted: (**a**) acute aquatic toxicity, expressed as the 96-h median lethal concentration (LC50, 96 h) toward the fathead minnow (*Pimephales promelas*); (**b**) bioconcentration factor; (**c**) developmental toxicity; and (**d**) mutagenicity.

To evaluate the environmental safety of the Fe_3_O_4_@MS/PMS treatment process for TC degradation, a quantitative toxicity assessment of the parent compound and its identified degradation intermediates was conducted using the Toxicity Estimation Software (Version 5.1.2) Tool (T.E.S.T.) developed by the U.S. Environmental Protection Agency (EPA). The U.S. EPA T.E.S.T. predictions were intended as a first-tier, screening-level (in silico) assessment of the relative hazard of TC versus its identified transformation products, following the widely used QSAR-based practice in AOP degradation-pathway studies. Its value lies in flagging intermediates that may be more hazardous than the parent compound information that directs subsequent experimental testing. The analysis focused on four key toxicity endpoints: acute aquatic toxicity (LC50-96h, fathead minnow (*Pimephales promelas*)), bioconcentration factor, developmental toxicity, and mutagenicity. The structures of the identified intermediates (from Section 3.9, Table S9) were optimized prior to toxicity prediction, and the results are summarized in Table S10. The temporal evolution of toxicity during the degradation process is illustrated in Figure 8. Toxicity analysis via T.E.S.T. software demonstrated that the Fe_3_O_4_@MS/PMS treatment process effectively reduces the environmental hazard of TC-contaminated water. The LC50-96h values increased from 3.33 mg/L (parent TC, “toxic”) to 274.16 mg/L (P9, “not harmful”), representing an 81.33-fold reduction in acute aquatic toxicity. The bioconcentration factor decreased from 4.91 L/kg (parent TC) to 1.08 L/kg (60 min), a 78.41% reduction indicating negligible bioaccumulation potential. Most importantly, mutagenicity was completely eliminated during the degradation process, with the ring-opening intermediates showing no toxicological concern. The temporal evolution of toxicity correlated with the degradation pathways identified by LC-MS, demonstrating that the formation of ring-opening and deamination products is critical for detoxification. These findings confirm that the Fe_3_O_4_@MS/PMS system produces a non-toxic effluent suitable for discharge or reuse, supporting its potential as a sustainable technology for antibiotic wastewater treatment. While the hydrolysis half-life of TC ranges from several days to weeks under typical conditions, substantially longer than the reaction time used in this study, the presence of these naturally occurring degradation products in receiving waters could modulate background toxicity. Studies have shown that photoproduct mixtures in natural waters can retain ecotoxicity even after the near-complete degradation of the parent compound, and that toxicity can increase with irradiation time in some cases. Therefore, while our treatment process effectively reduces the concentration of the parent antibiotic and produces less toxic intermediates, the potential contribution of naturally occurring TC degradation products to overall toxicity in receiving waters should be considered in a comprehensive risk assessment. Future studies should investigate the combined toxicity of treatment-generated and naturally occurring degradation products to fully assess the environmental safety of treated effluents.

### 3.11. Life Cycle Assessment

**Figure 9:**
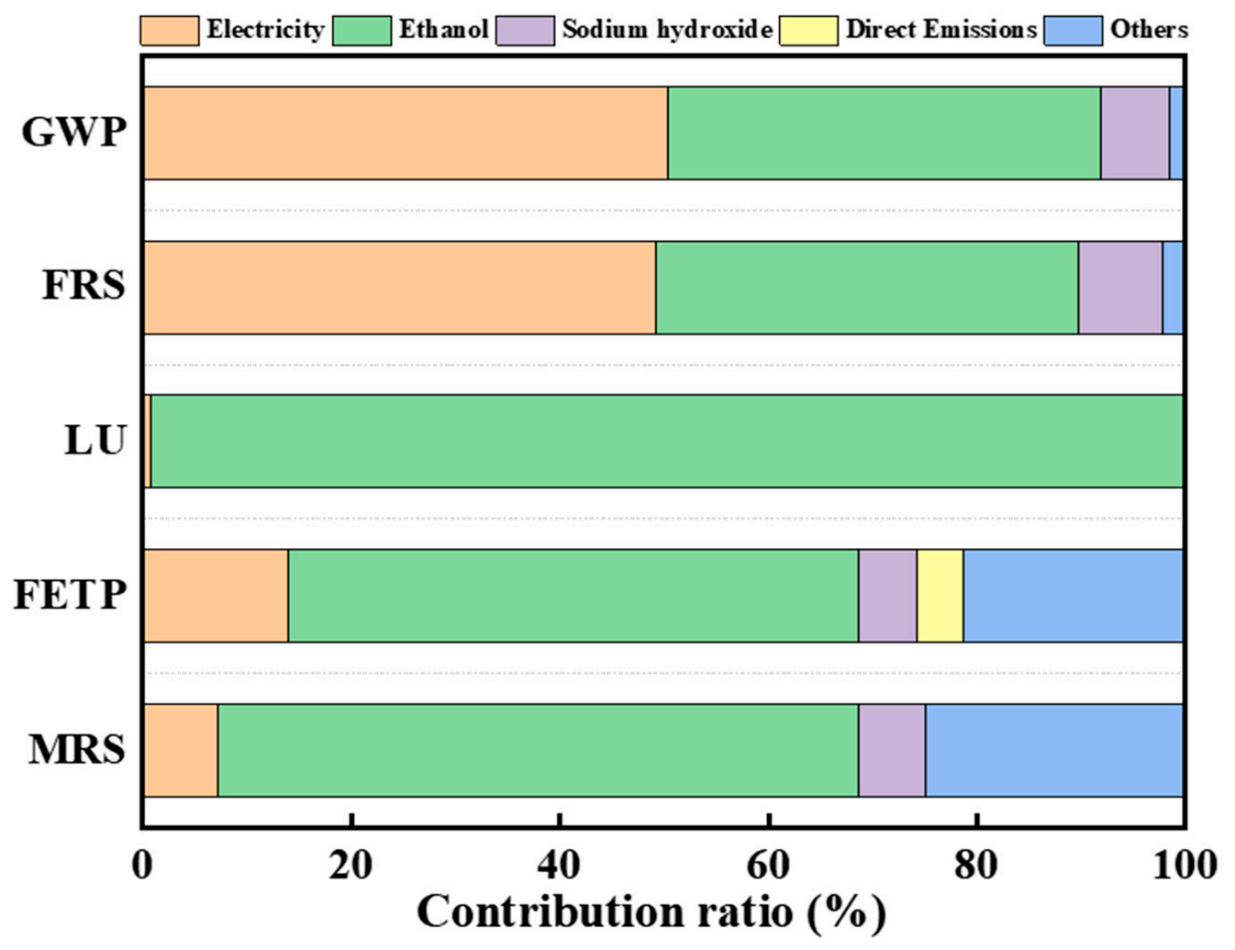
Environmental hotspot analysis of the cradle-to-gate production of the Fe_3_O_4_@MS catalyst, calculated with the open-source life cycle assessment (LCA) software openLCA (version 2.6.2). A “hotspot” denotes the process step or input contributing the largest share to a given impact category. Impact categories shown: GWP—global warming potential; FRS—Fossil Resource Scarcity; LU—Land Use; FETP—Freshwater Ecotoxicity Potential; MRS—Mineral Resource Scarcity.

To identify the primary environmental burdens associated with the production of the Fe_3_O_4_@MS composite, a contribution analysis was performed across the five selected impact categories, as illustrated in Figure 9. Overall, ethanol consumption and electricity usage are the dominant environmental hotspots. Specifically, ethanol accounts for nearly 100% of the LU impact. This is primarily attributed to the significant agricultural land required for cultivating biomass crops (such as corn or sugarcane) used in fermentation-based ethanol production. Regarding GWP and FRS, the impacts are almost equally shared between electricity (~50%) and ethanol (~40%). The substantial electricity contribution is driven by energy-intensive stages such as high-temperature alkali fusion and calcination. These processes rely heavily on the national grid, which remains dominated by fossil-fuel-based power generation in the Chinese context [70,71,72].

The analysis of MRS provides a compelling argument for waste valorization. Ethanol (~61%) and the “Others” category (~25%, comprising reagents like H_2_SO_4_ and the P123 template) are the main contributors, followed by sodium hydroxide (~6%). Crucially, the primary raw material—CSS—contributes zero burden to MRS. This follows the “cut-off” rule for industrial solid waste, validating the significant environmental benefit of upcycling metallurgical byproducts into functional materials, thereby effectively avoiding the depletion of virgin mineral ores.

Furthermore, the FETP reveals a nuanced impact distribution. While upstream indirect impacts are dominated by ethanol (~55%) and the “Others” category (~21%), Direct Emissions (representing the wastewater containing residual salts and organics) contribute approximately 4.36% specifically to this category. This precise capture of direct ecotoxicity, even at a relatively low percentage, underlines the rigor of the established system boundary and the accuracy of the laboratory-to-inventory scaling.

The LCI and contribution analysis clearly indicate that the massive use of organic solvents and high energy consumption are the primary bottlenecks for the environmental sustainability of Fe_3_O_4_@MS production. However, as integrated into the system boundary (Figure 1), the implementation of an ethanol recovery loop during the washing phase serves as a critical mitigation strategy. By recycling the solvent, the predominant burdens on LU, GWP, and FRS can be drastically reduced. This transition from a linear “disposal” model to a circular “recovery” model further highlights the green chemistry principles embedded in this waste-to-resource valorization process.

### 3.12. Process Comparison, Biodegradability and Outlook Toward Experimental Risk Assessment and Practical Engineering Considerations

Compared with established chemical oxidation technologies (see Table S11), the Fe_3_O_4_@MS/PMS process offers several practical advantages: the catalyst is derived from copper smelting slag, coupling solid-waste valorization with water treatment; PMS activation operates efficiently over a wider pH range than the classical Fenton reaction; the magnetic catalyst can be readily recovered and reused; and no external light or electrical energy input is required. On the other hand, the process consumes a chemical oxidant and introduces sulfate residuals, its performance in complex real-water matrices and over long-term operation remains to be validated, mineralization is incomplete, and demonstration at larger scale is still pending. Ozonation, although mature, entails high energy demand for ozone generation and potential bromate formation; Fenton-based processes are constrained to acidic pH and produce iron sludge; photocatalysis requires irradiation and suffers from limited solar utilization.

Biological treatment, the workhorse of wastewater purification, is of limited efficacy for TC. Under the OECD/ISO biodegradation testing framework—the gold standard for such assessments—TC is generally not readily biodegradable in OECD 301-type screening tests (and their ISO equivalents), largely because its antibacterial activity inhibits the non-acclimated inocula; in higher-tier inherent (OECD 302), activated-sludge simulation (OECD 303) and environmental simulation (OECD 308/309) tests, removal is frequently dominated by sorption rather than mineralization [73]. This recalcitrance justifies oxidative pretreatment: AOPs and biological treatment are complementary, and the biodegradability of the oxidation effluent (e.g., BOD5/COD evolution or an OECD 301 test on the treated solution) is a relevant endpoint for process combination.

Finally, the in-silico toxicity evaluation performed with the U.S. EPA T.E.S.T. software (Section 3.10) must be regarded as a screening-level tool: all quantitative structure–activity relationship (QSAR)-derived values carry model uncertainty and cannot substitute for experimental biotests. A final risk assessment of the proposed method therefore requires standardized experimental testing across trophic levels, following the OECD/ISO framework [74]: bacterial luminescence inhibition with “Aliivibrio fischeri” (Microtox, ISO 11348-3 [75]), algal growth inhibition (OECD 201), acute and chronic tests with “Daphnia magna” (OECD 202/211), and activated sludge respiration inhibition (OECD 209) to verify compatibility with biological post-treatment. Such a test battery, applied to samples along the degradation timeline, will be the core of a subsequent follow-up study.

For practical implementation of the Fe_3_O_4_@MS/PMS system, three key engineering aspects must be considered: catalyst introduction equipment, integration into treatment schemes, and spent catalyst disposal. Regarding equipment, a stirred-tank reactor (STR) coupled with an external separation unit was recommend as the most practical and proven approach for introducing and recovering the Fe_3_O_4_@MS catalyst, achieving complete retention and reuse over multiple cycles. Alternatively, a fluidized bed reactor (FBR) can be employed for continuous-flow operations. Regarding integration into treatment schemes, the Fe_3_O_4_@MS/PMS system is best positioned as a pre-treatment stage upstream of biological treatment for industrial or hospital wastewater containing antibiotics. This configuration eliminates the antibacterial activity of TC before it reaches the biological community, protecting the downstream treatment process. For municipal wastewater treatment, the system can serve as an advanced polishing step after secondary biological treatment to remove residual micropollutants. As for spent catalyst disposal, a hierarchical approach is recommended: (i) regeneration to extend catalyst lifespan; (ii) resource recovery through acid leaching of iron and repurposing of silica; and (iii) safe disposal in non-hazardous waste landfills.

## 4. Conclusions

In this study, a novel Fe_3_O_4_@MS catalyst was successfully synthesized from CSS via an alkali fusion–hydrothermal method and applied for PMS activation to degrade TC. The following conclusions can be drawn:

(1) The Fe_3_O_4_@MS was successfully prepared with well-dispersed Fe_3_O_4_ particles uniformly anchored on the MS surface. The Fe_3_O_4_@MS/PMS system achieved 98.70% TC removal within 60 min under optimized conditions (catalyst 0.5 g/L, PMS 1.0 mmol/L, initial TC concentration 50 mg/L, initial pH 6.5, 25 °C).
(2) TC degradation followed pseudo-first-order kinetics. The reaction orders were determined as α = 0.7433 for catalyst dosage, β = 1.0149 for PMS concentration, and γ = −0.6146 for initial TC concentration. The activation energy was calculated to be 27.04 kJ/mol, indicating a surface-mediated chemical reaction.
(3) The Fe_3_O_4_@MS/PMS system exhibited excellent TC degradation performance across a broad pH range of 3.0–13.0 and showed good tolerance to common anions. Moreover, the Fe_3_O_4_@zeolite catalyst exhibited remarkable stability, retaining over 90% after five consecutive cycles, with low iron leaching.
(4) Radical quenching and EPR spectroscopy revealed that multiple ROS contributed to TC degradation, with •OH being the dominant species (60.14%), followed by ^1^O_2_ (54.17%), O_2_^•−^ (34.78%), and SO_4_^•−^ (23.47%). The non-radical ^1^O_2_ played a significant role, facilitated by the confinement effect of the mesoporous structure.
(5) Nine degradation intermediates were identified by LC-MS, leading to three proposed pathways: (I) hydroxylation and demethylation, (II) ring-opening and quinone formation, and (III) deamination and mineralization. T.E.S.T. analysis on the intermediates demonstrated that the Fe_3_O_4_@MS/PMS treatment significantly reduced environmental hazards.
(6) LCA revealed that the massive use of organic solvents and high energy consumption are the primary bottlenecks for the environmental sustainability of Fe_3_O_4_@MS production. However, by recycling the solvent, the predominant burdens on LU, GWP, and FRS can be drastically reduced. This transition model further highlights the green chemistry principles embedded in this waste-to-resource valorization process.

In summary, this study demonstrates that CSS can be valorized into a high-performance Fe_3_O_4_@MS catalyst for PMS activation, achieving efficient degradation and detoxification of TC. The combination of waste-derived catalyst synthesis, excellent catalytic performance, multiple ROS generation, low toxicity of degradation products, favorable life cycle impacts, and good reusability positions this system as a promising and sustainable technology for antibiotic wastewater treatment. The findings provide a theoretical and practical foundation for the application of industrial solid waste-derived catalysts in AOPs, contributing to both waste management and water remediation within a circular economy framework. In the future work, the screening-level QSAR toxicity predictions presented here should be superseded by experimental ecotoxicity testing of TC and its transformation products using a standardized OECD/ISO biotest battery. And the biodegradability of the oxidation effluent (e.g., BOD5/COD evolution and OECD 301-type tests on treated solutions) should be evaluated to design and verify the coupling of the AOP pretreatment with biological post-treatment. Together, these studies will pave the way toward the practical application of slag-derived Fe_3_O_4_@MS/PMS technology for antibiotic removal from water.

## Data Availability

The raw data supporting the conclusions of this article will be made available by the authors on request.

## Supplementary Materials

The following supporting information can be downloaded at: https://www.mdpi.com/article/10.3390/toxics14090757/s1, Figure S1. XRD patterns of the Fe_3_O_4_@MS catalysts; Figure S2. SEM images of the synthesized Fe_3_O_4_@MS; Figure S3. SEM-EDS elemental mapping of the synthesized Fe_3_O_4_@MS; Figure S4. (a) N_2_ adsorption–desorption isotherm plot and (b) pore size distribution of Fe_3_O_4_@MS; Figure S5. ln(k_obs_) versus ln[Catalyst]; Figure S6. ln(k_obs_) versus ln[PMS]; Figure S7. ln(k_obs_) versus ln[TC]; Figure S8. lnk versus 1/T; Figure S9. ln(k/T) versus 1/T; Figure S10. (a) Reusability and (b) leaching concentration of Fe during TC degradation by Fe_3_O_4_@MS/PMS over five degradation cycles; Figure S11. The pseudo-first-order fitting under different scavengers; Figure S12. Fukui function equivalence surface for TC molecules; Table S1. LCI for the production of 1 kg of Fe_3_O_4_@MS catalyst; Table S2. TC degradation efficiency under different heterogeneous catalysts for PMS activation [10,25,27,68,76,77,78,79,80]; Table S3. The calculated kinetic parameters under different catalyst dosage; Table S4. The calculated kinetic parameters under different PMS concentration; Table S5. The calculated kinetic parameters under different initial TC concentration; Table S6. The calculated kinetic parameters under different temperature; Table S7. The calculated kinetic parameters under different scavengers; Table S8. Calculated values of electrophilicity (f^−^), radicals (f^0^) and nucleophilicity (f^+^) for TC; Table S9. Information on intermediate products; Table S10. Information on intermediates’ ecotoxicity indicators; Table S11. The comparison with other chemical oxidation methods.

## Abbreviations

The following abbreviations are used in this manuscript:

AOPs	Advanced Oxidation Processes
BET	Brunauer–Emmett–Teller
BJH	Barrett–Joyner–Halenda
BMPO	5-tert-Butoxycarbonyl-5-methyl-1-pyrroline N-oxide
CSS	Copper Smelting Slag
DFT	Density Functional Theory
DMPO	5,5-Dimethyl-1-pyrroline N-oxide
EPR	Electron Paramagnetic Resonance
EDS	Energy dispersive spectroscopy
FTIR	Fourier Transform Infrared Spectroscopy
FRS	Fossil Resource Scarcity
FETP	Freshwater Ecotoxicity Potential
FBR	Fluidized bed reactor
GWP	Global Warming Potential
IC	Ion chromatography
LC-MS	Liquid Chromatography–Mass Spectrometry
LCA	Life Cycle Assessment
L-His	L-histidine
LU	Land Use
MS	Mesoporous Silica
MeOH	Methanol
MRS	Mineral Resource Scarcity
PMS	Peroxymonosulfate
p-BQ	p-benzoquinone
QSAR	Quantitative Structure–Activity Relationship
ROS	Reactive Oxygen Species
SEM	Scanning Electron Microscopy
STR	Stirred-tank reactor
TC	Tetracycline
TBA	Tert-butyl alcohol
TEMP	2,2,6,6-Tetramethyl-4-piperidinol
T.E.S.T.	Toxicity Estimation Software Tool
TOC	Total Organic Carbon
XPS	X-ray Photoelectron Spectroscopy
XRD	X-ray Diffraction
