# Supplementary material for: Copper Smelting Slag-Derived Fe3O4@Mesoporous Silica for Peroxymonosulfate Activation and Tetracycline Degradation: Performance, Mechanism, and Life Cycle Assessment
Source: Toxics. 2026 Aug 26;14(9):757. doi: 10.3390/toxics14090757 (PMC13611212; doi:10.3390/toxics14090757)
Supplement: Supplementary file 1 [file toxics-14-00757-s001.zip › toxics-4492139-supplementary.pdf]

Supplementary Material

Copper smelting slag-derived Fe_3O_4@mesoporous silica for peroxymonosulfate activation and tetracycline degradation: Performance, mechanism, and life cycle assessment

Changxin Li*, Xiaoya Li, Jinyu Yang, Nan Liu, Shanpei Liu, Xianglong Huang, Huaxin Zhang

Hubei Key Laboratory of Drug Synthesis and Optimization, Jingchu University of Technology, Jingmen 448000, China

*** Corresponding author:**

Xiangshan Road 33#, Dongbao District, Jingmen, Hubei, 448000, China.

Email: lll07060258@163.com

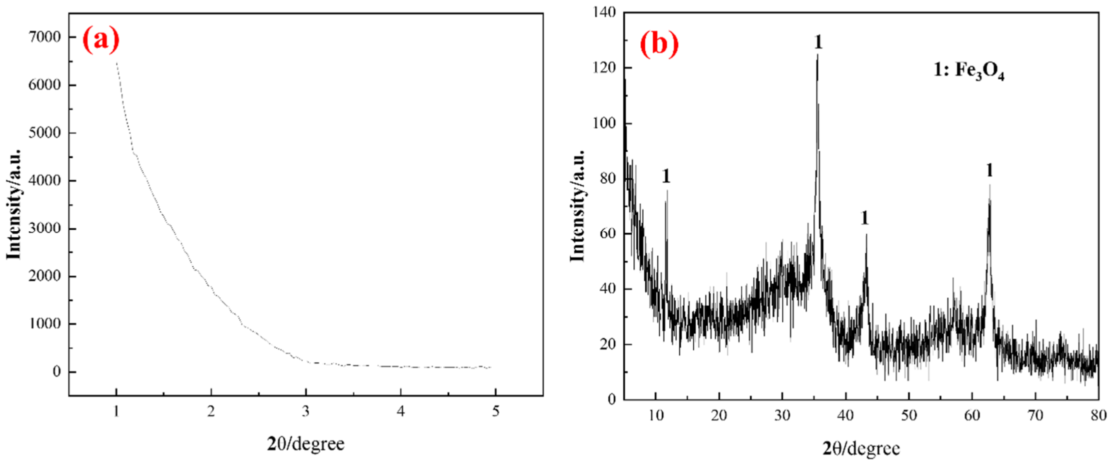

Figure S1. XRD patterns of the Fe_3O_4@MS catalysts.

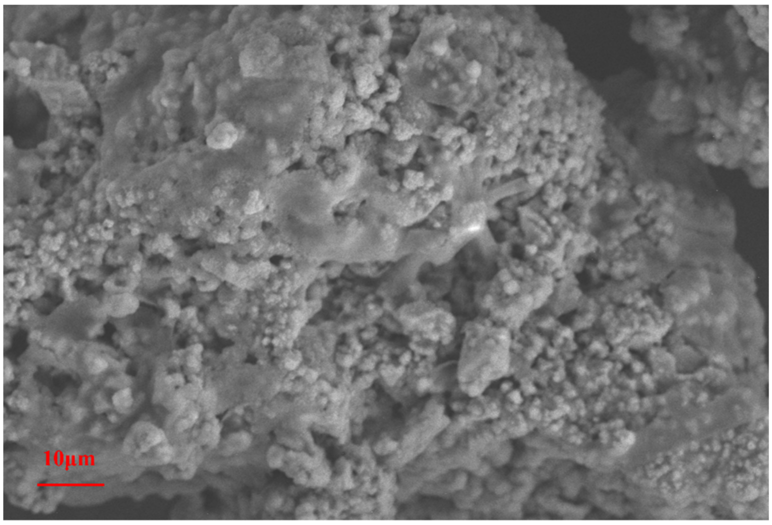

Figure S2. SEM images of the synthesized Fe_3O_4@MS

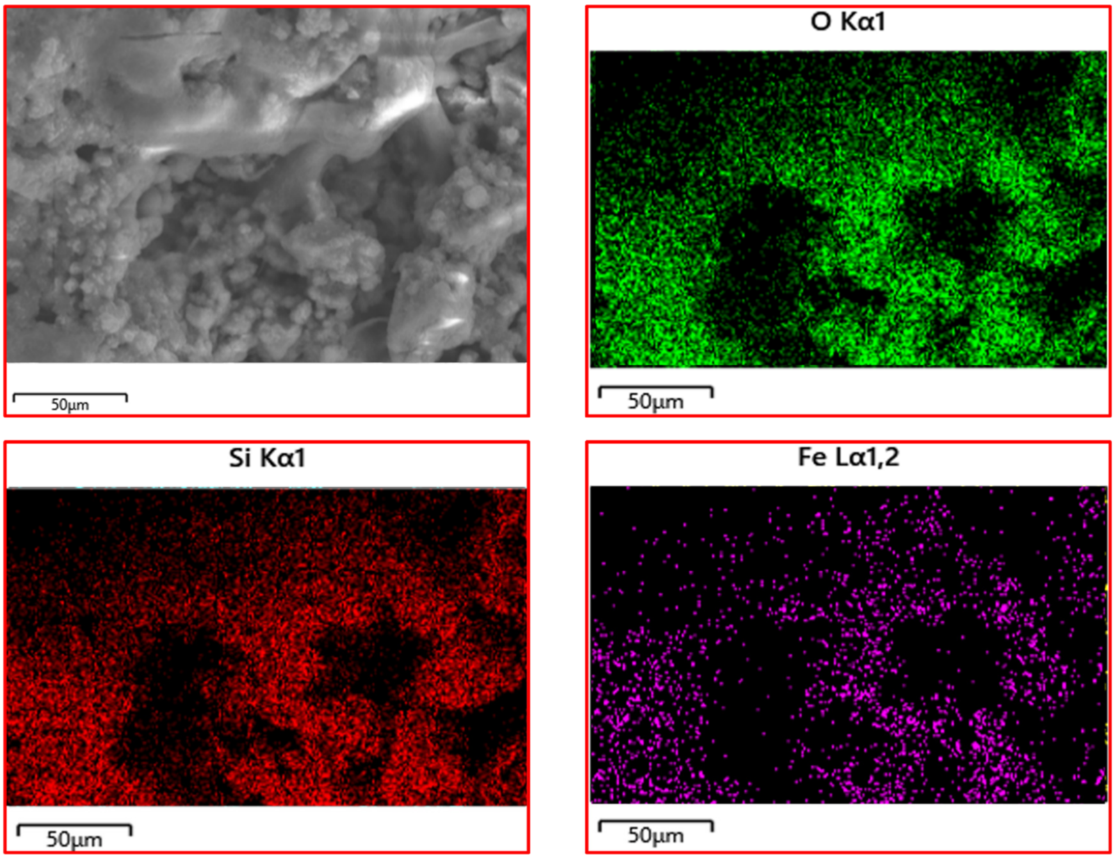

Figure S3. SEM-EDS elemental mapping of the synthesized Fe_3O_4@MS

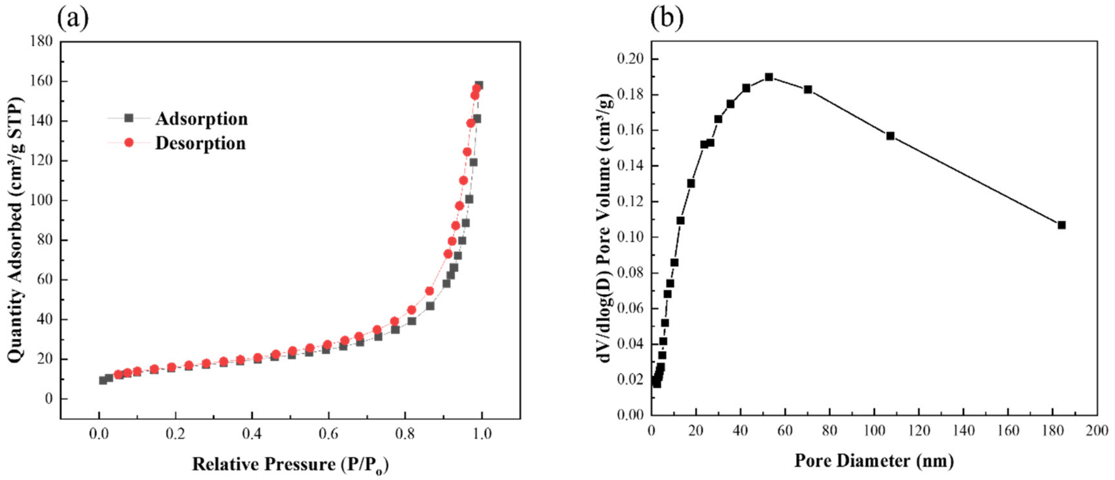

Figure S4. (a) N_2 adsorption-desorption isotherm plot and (b) pore size distribution of Fe_3O_4@MS.

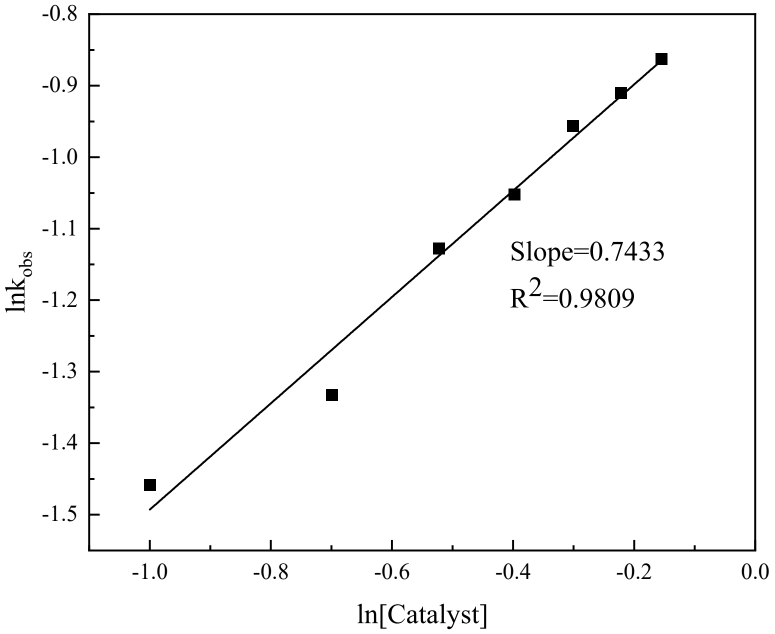

Figure S5. ln(k_{obs}) versus ln[Catalyst].

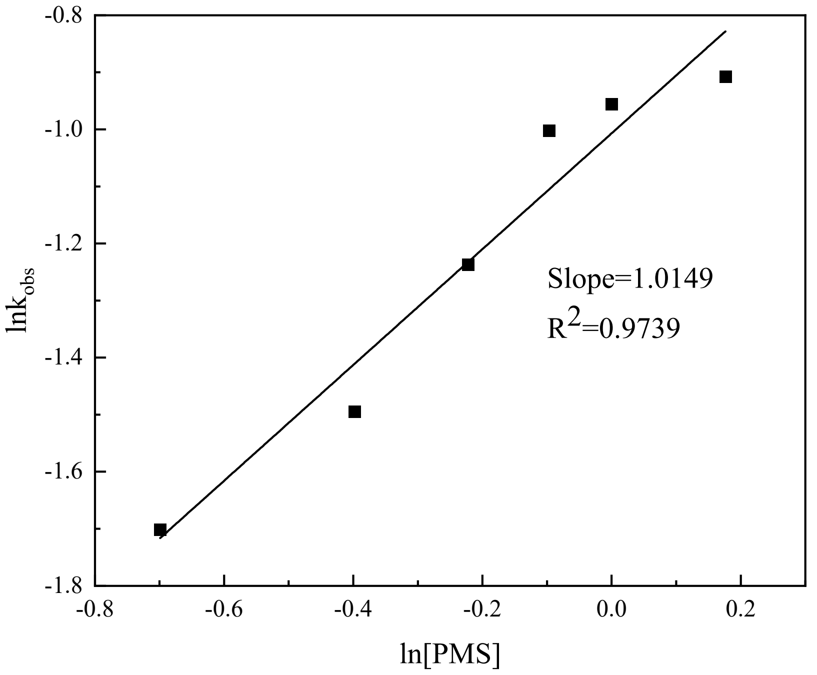

Figure S6. ln(k_{obs}) versus ln[PMS].

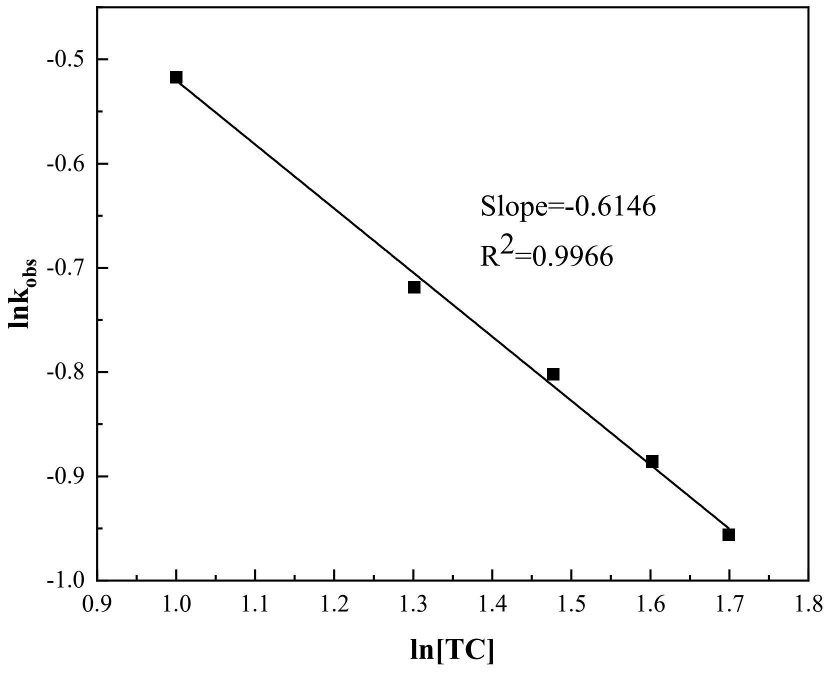

Figure S7. ln(k_{obs}) versus ln[TC].

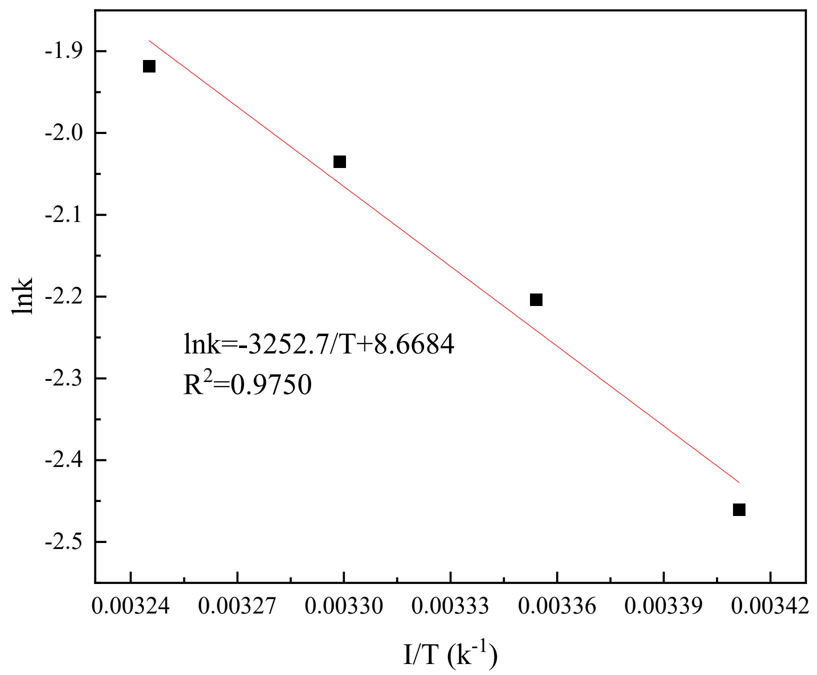

Figure S8. lnk versus 1/T.

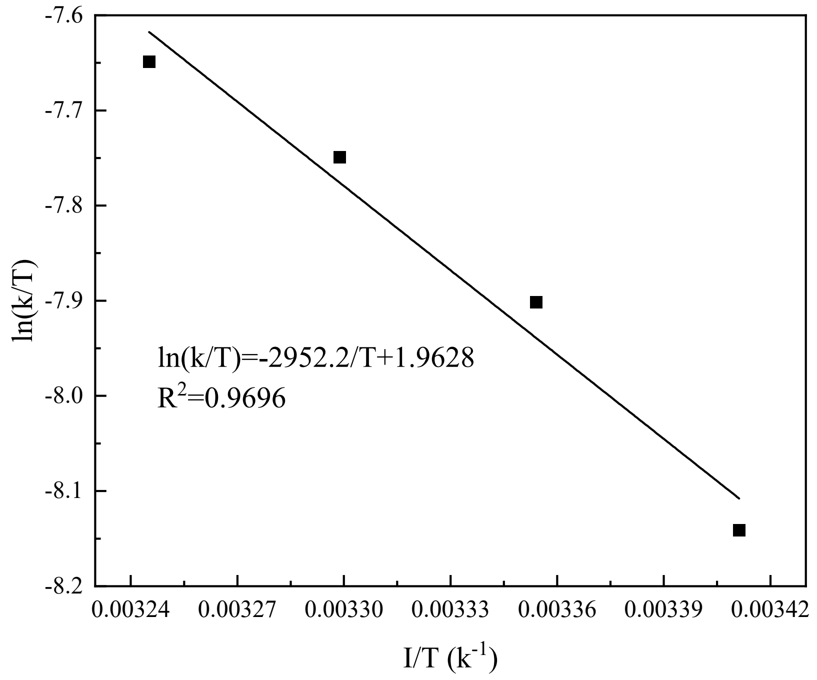

Figure S9. ln(k/T) versus 1/T.

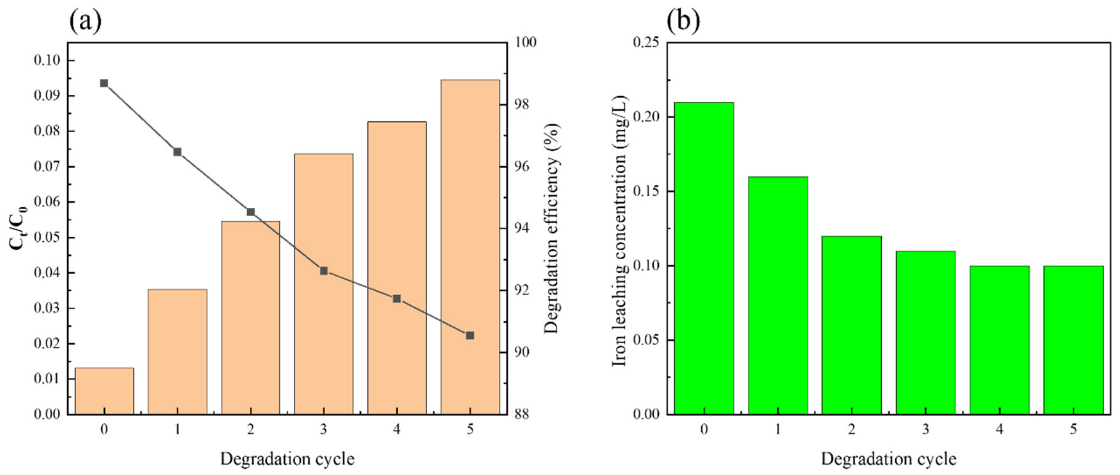

Figure S10. (a) Reusability and (b) leaching concentration of Fe during TC degradation by Fe_3O_4@MS/PMS over five degradation cycles. (Experimental conditions: TC concentration = 50 mg/L, PMS concentration = 1.0 mmol/L, catalyst dosage = 0.5 g/L, reaction time = 60 min, temperature = 25 °C and initial pH = 6.8).

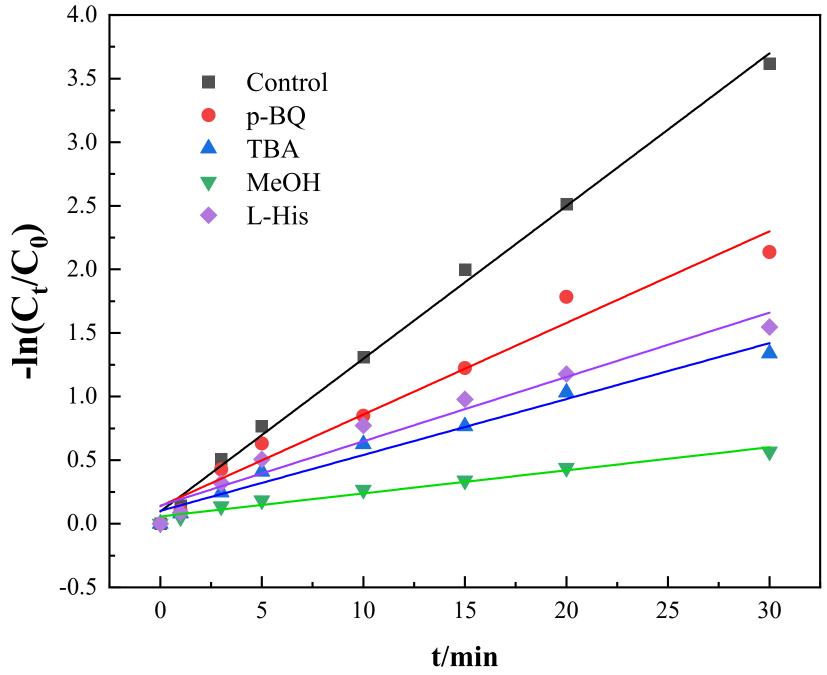

Figure S11. The pseudo-first-order fitting under different scavengers (Experimental conditions: TC concentration = 50 mg/L, PMS concentration = 1.0 mmol/L, catalyst dosage = 0.5 g/L, initial pH = 6.8, temperature = 25 °C).

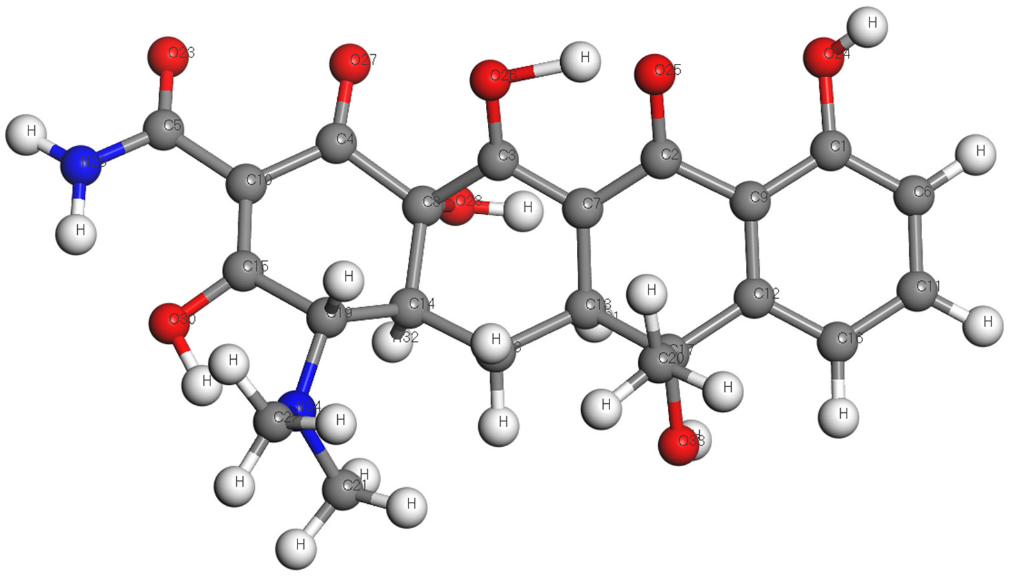

Figure S12. Fukui function equivalence surface for TC molecules.

Table S1. LCI for the production of 1 kg of Fe_3O_4@MS catalyst.

**Inputs/Outputs**	**Substance / Energy**	**Unit**	**Amount**	**Data Source / Background Process**
Inputs				
	CSS	kg	1.32	Zero-burden assumption; only transport is accounted for (Transport, freight, lorry>32 metric ton, EURO4 {GLO})
	Sodium hydroxide (NaOH)	kg	1.71	Sodium hydroxide, without water, in 50% solution state {GLO} | market for
	Sulfuric acid (H_2SO_4)	kg	2.17	Sulfuric acid {GLO} | market for
	P123 template	kg	0.026	Polyethylene glycol {GLO} | market for
	Ethanol (anhydrous)	kg	16.6	Ethanol, without water, in 99.7% solution state, from fermentation {GLO} | market for
	Deionized water	kg	92.1	Water, deionized, from tap water, at user terminal {GLO} | market for
	Electricity	kWh	20.0	Chinese medium-voltage electricity market. Conservative industrial benchmark adjustment
Outputs				
	Fe_3O_4@MS catalyst	kg	1	Functional unit of this study
	Waste ethanol	kg	16.6	Modeled in Water compartment as Ethanol (Assuming 100% of washing ethanol enters the waste liquid)
	Sodium sulfate (Na_2SO_4)	kg	3.14	Modeled as Sodium sulfate (or separately as Sodium, ion and Sulfate)
	Waste water	kg	94.0	Recorded as physical flow to maintain mass balance (Excluded from LCIA to avoid artifactual water credits)

Note: For the purpose of clarity in the subsequent contribution analysis, inputs with minimal environmental burdens, specifically sulfuric acid, P123 template, deionized water, and transport, are aggregated into an “Others” category.

Table S2. TC degradation efficiency under different heterogeneous catalysts for PMS activation.

Catalyst	PMS concentration (mmol/L)	Catalyst dose (g/L)	TC concentration (mg/L)	*T* (°C)	Degradation efficiency (%)	Ref.
CFO/CN	2.00	0.20	40	20	86.80%	[1]
Fe-BC	0.60	0.80	50	25	86.19%	[2]
Fe_5-NG	0.50	0.10	25	25	100%	[3]
Fe@BC	6.00	0.80	20	25	90.00%	[4]
Co_3O_4/CPANI	1.00	0.15	20	25	92.11%	[5]
MoS_2	1.00	0.05	20	20	94.20%	[6]
FMDS-3	3.00	0.20	40	22	90.70%	[7]
B-NPC	0.44	0.04	20	25	100.0%	[8]
Fe-N/BC	0.56	0.05	30	25	83.90%	[9]
Fe_3O_4@MS	1.00	0.50	50	25	98.70%	This work

Table S3. The calculated kinetic parameters under different catalyst dosage.

Catalyst dosage (g/L)	K_{obs}(min^{-1})	R^2	Standard error ($\times10^{-3}$)	Degradation efficiency (%)	$t_{1/2}$ (min)
0.1	0.0348	0.9604	1.12	78.80	19.92
0.2	0.0465	0.9724	2.02	86.97	14.91
0.3	0.0746	0.9960	0.98	94.60	9.29
0.4	0.0887	09972	1.83	98.29	7.81
0.5	0.1107	0.9903	2.09	98.69	6.26
0.6	0.1231	0.9913	1.87	99.33	5.63
0.7	0.1372	0.9914	2.59	99.68	5.05

Note: Experimental conditions: TC concentration = 50 mg/L, PMS concentration = 1.0 mmol/L,

initial pH = 6.8, temperature = 25 °C. Degradation efficiency obtained at a reaction time of 60 min. k_{obs} is the pseudo-first-order rate constant. $t_{1/2}$ (min) represents the half-life calculated as ln(2)/k_{obs}. Data are presented as mean values from triplicate experiments.

Table S4. The calculated kinetic parameters under different PMS concentration.

PMS concentration (mmol/L)	K_{obs}(min^{-1})	R^2	Standard error ($\times10^{-3}$)	Degradation efficiency (%)	$t_{1/2}$ (min)
0.2	0.0199	0.9824	1.82	57.20	34.83
0.4	0.032	0.9819	2.68	78.80	21.66
0.6	0.0579	0.9764	3.08	89.42	11.97
0.8	0.0994	09865	1.93	97.88	6.97
1.0	0.1107	0.9903	2.09	98.69	6.26
1.5	0.1236	0.9794	2.17	99.18	5.61

Note: Experimental conditions: TC concentration = 50 mg/L, catalyst dosage = 0.5 g/L, initial pH = 6.8, temperature = 25 °C. Degradation efficiency obtained at a reaction time of 60 min. kobs is the pseudo-first-order rate constant. $t_{1/2}$ (min) represents the half-life calculated as ln(2)/k_{obs}. Data are presented as mean values from triplicate experiments.

Table S5. The calculated kinetic parameters under different initial TC concentration.

Initial TC concentration (mg/L)	K_{obs}(min^{-1})	R^2	Standard error ($\times10^{-3}$)	Degradation efficiency (%)	$t_{1/2}$ (min)
10	0.3042	0.9772	2.07	99.89	2.28
20	0.1911	0.9847	2.96	99.73	3.63
30	0.1579	0.9851	2.64	99.37	4.39
40	0.1301	09932	1.69	98.98	5.33
50	0.1107	0.9903	2.09	98.69	6.26

Note: Experimental conditions: PMS concentration = 1.0 mmol/L, catalyst dosage = 0.5 g/L, initial

pH = 6.8, temperature = 25 °C. Degradation efficiency obtained at a reaction time of 60 min. kobs is the pseudo-first-order rate constant. $t_{1/2}$ (min) represents the half-life calculated as $\ln(2)/k_{obs}$. Data are presented as mean values from triplicate experiments.

Table S6. The calculated kinetic parameters under different temperature.

Temperature (°C)	K_{obs}(min⁻¹)	R^2	Standard error (×10⁻³)	Degradation efficiency (%)	$t_{1/2}$ (min)
20	0.0854	0.9723	1.87	95.68	8.12
25	0.1107	0.9903	2.37	98.69	6.26
30	0.1307	0.9940	2.29	99.58	5.30
35	0.1469	09719	3.09	99.78	4.72

Note: Experimental conditions: TC concentration = 50 mg/L, PMS concentration = 1.0 mmol/L, catalyst dosage = 0.5 g/L, initial pH = 6.8. Degradation efficiency obtained at a reaction time of 60 min. kobs is the pseudo-first-order rate constant. $t_{1/2}$ (min) represents the half-life calculated as $\ln(2)/k_{obs}$. Data are presented as mean values from triplicate experiments.

Table S7. The calculated kinetic parameters under different scavengers.

Scavenger	K_{obs}(min⁻¹)	R^2	Standard error (×10⁻³)	Degradation efficiency (%)	$t_{1/2}$ (min)	CR_{ROS} (%)
Control	0.1104	0.9903	2.37	98.69	6.26	0
p-BQ	0.0720	0.9707	2.86	89.50	9.63	34.78
TBA	0.0440	0.9749	1.98	73.80	15.75	60.14
MeOH	0.0181	0.9688	2.62	43.33	38.30	83.61
L-His	0.0506	0.9688	2.75	78.72	13.70	54.17

Note: Experimental conditions: TC concentration = 50 mg/L, PMS concentration = 1.0 mmol/L, catalyst dosage = 0.5 g/L, initial pH = 6.8, temperature = 25 °C. Degradation efficiency obtained at a reaction time of 60 min. k_{obs} is the pseudo-first-order rate constant. $t_{1/2}$ (min) represents the half-

life calculated as ln(2)/k_{obs}. CR_{ROS} is the contribution rate of each reactive species and was calculated using following equation. Data are presented as mean values from triplicate experiments.

$$CR_{ROS} = \frac{\text{kobs,control-kobs,scavenger}}{\text{kobs,control}} \times 100\%$$

Table S8. Calculated values of electrophilicity (f^-), radicals (f^0) and nucleophilicity (f^+) for TC.

Atom	f^-	f^0	f^+
C(1)	0.008	0.022	0.035
C(2)	0.014	0.044	0.073
C(3)	-0.012	0.03	0.071
C(4)	0.046	0.022	-0.002
C(5)	0.043	0.023	0.003
C(6)	0.013	0.025	0.037
C(7)	0	0.015	0.03
C(8)	0.023	0.015	0.006
C(9)	-0.002	0.01	0.023
C(10)	0.021	0.013	0.005
C(11)	0.018	0.043	0.069
C(12)	0.001	0.016	0.032
C(13)	0.001	0.002	0.003
C(14)	0.006	0.004	0.002
C(15)	0.042	0.024	0.006
C(16)	0.009	0.02	0.032
C(17)	0.001	0.001	0.001
C(18)	0.006	0.005	0.004
C(19)	0.006	0.003	0
C(20)	0.003	0.005	0.007

C(21)	0.006	0.004	0.003
C(22)	0.005	0.004	0.003
O(23)	0.168	0.09	0.012
O(24)	0.006	0.016	0.026
O(25)	0.023	0.046	0.07
O(26)	-0.006	0.039	0.084
O(27)	0.165	0.086	0.008
O(28)	0.011	0.022	0.034
N(29)	0.063	0.037	0.011
O(30)	0.042	0.03	0.018
H(31)	0.004	0.009	0.014
H(32)	0.008	0.01	0.012
O(33)	0.007	0.014	0.02
N(34)	0.005	0.003	0.001

Table S9. Information on intermediate products.

Compound	Molecular formula	M/Z
TC	$C_{22}H_{24}N_2O_8$	445
P1	$C_{21}H_{22}N_2O_7$	415
P2	$C_7O_5N_3H_{11}$	383
P3	$C_{21}H_{20}O_7$	347
P4	$C_{17}H_{16}O_4$	283
P5	$C_{15}H_{18}O_3$	247
P6	$C_{11}H_{12}O_2$	175
P7	$C_{10}H_{12}O$	148
P8	$C_7H_{12}O_2$	129
P9	$C_4H_6O_3$	102

Table S10. Information on intermediates' ecotoxicity indicators.

Compound	LC_{50}-96h ($mg \cdot L^{-1}$)	Bioconcentration factor	Developmental toxicity	Mutagenicity
TC	3.33	4.91	0.85	1.00
P1	0.38	0.47	0.84	0.37
P2	1.28	4.91	0.79	0.59
P3	0.15	28.66	0.99	0.19
P4	2.25	1.69	1.06	0.17
P5	9.06	13.29	0.71	0.25
P6	12.37	12.40	0.82	0.39
P7	3.65	13.08	0.74	0.04
P8	126.98	1.66	0.66	0.04
P9	274.16	1.08	0.67	0.14

Table S11. The comparison with other chemical oxidation methods

Technology	Oxidant / driver	Strengths	Weaknesses
Ozonation	O_3	Strong oxidant, no residual chemical, mature at full scale	High energy cost of O_3 generation, selective reactivity, possible bromate formation, poor mineralization
Fenton/electro-Fenton	$H_2O_2 + Fe^{2+}$	Simple, fast	Narrow pH (~3), iron sludge production, H_2O_2 handling
Photocatalysis (e.g., TiO_2)	Light + catalyst	No consumable oxidant	Light demand, low solar efficiency, catalyst recovery

H_2O_2-based AOPs (UV/H_2O_2)	H_2O_2 + UV	Clean reagents	UV energy input, limited radical yield
PMS activation by Fe_3O_4@MS (this work)	PMS + solid catalyst	Wide pH range, high redox potential and selectivity, waste-derived catalyst	Oxidant cost

References

1. Pan, B.; Chen, W.; Zhou, L.X.; Lai, X.J.; Qin, J.N.; Wang, C.Y. $CoFe_2O_4$/Carbon Nitride Z-Scheme Heterojunction Photocatalytic PMS Activation for Efficient Tetracycline Degradation: Accelerated Electron Transfer. *Process Saf. Environ. Prot.* **2024**, *191*, 2522–2532, doi:10.1016/j.psep.2024.09.120.
2. You, Y.X.; Song, L.; Guo, Y.; Zhang, X.J.; Hu, S. Coupled Homogeneous-Heterogeneous Fenton-like System to Highly Efficiently Degrade Tetracycline in Water at Circumneutral pH: Mechanism and Toxicity Evaluation. *J. Water Process Eng.* **2025**, *72*, 107569, doi:10.1016/j.jwpe.2025.107569.
3. Wang, S.H.; Song, Y.H.; Wu, Y.S.; Wang, B.; Gao, X.; Zhu, X.W.; Liu, J.Y.; Chu, P.K. Degradation of Tetracycline by Fe-N-coordinated Porous Carbon Activated PMS: High Dispersibility and Stability. *Adv. Sustainable Syst.* **2025**, *9*, 2500219, doi:10.1002/adsu.202500219.
4. Yu, F.K.; Song, Y.; Guo, Y.P.; Yang, J. Degradation of Tetracycline by Persulfate Oxidation Promoted by Iron-Modified Biochar. *New J. Chem.* **2022**, *46*, 22459–22468, doi:10.1039/D2NJ03348H.
5. Qian, J.; Mi, X.H.; Chen, Z.J.; Xu, W.H.; Liu, W.; Ma, R.; Zhang, Y.C.; Du, Y.F.; Ni, B.J. Efficient Emerging Contaminants (EM) Decomposition via Peroxymonosulfate (PMS) Activation by Co_3O_4/Carbonized Polyaniline (CPANI) Composite: Characterization of Tetracycline (TC) Degradation Property and Application for the Remediation of EM-Polluted Water Body. *J. Cleaner Prod.* **2023**, *405*, 137023, doi:10.1016/j.jclepro.2023.137023.

6. Yan, Z.L.; He, F.; Zhang, J.Y.; Fang, J.R.; Wang, J.J.; Zhou, H.H. Enhanced Fe(III)/PMS System by Flower-like MoS_2 Nanosheet for Rapid Degradation of Tetracycline. *J. Environ. Chem. Eng.* **2022**, *10*, 108860, doi:10.1016/j.jece.2022.108860.
7. Chen, P.; Cheng, Z.L.; Zhang, X.Z.; Yan, C.Q.; Wei, J.; Qiu, F.C.; Liu, Y. Fe–Mn Bimetallic Catalyst to Activate Peroxymonosulfate (PMS) for Efficient Degradation of Tetracycline: Mechanism Insights and Application for Pharmaceutical Wastewater. *J. Cleaner Prod.* **2024**, *445*, 141365, doi:10.1016/j.jclepro.2024.141365.
8. Xie, J.L.; Luo, X.; Chen, L.; Gong, X.B.; Zhang, L.R.; Tian, J. ZIF-8 Derived Boron, Nitrogen Co-Doped Porous Carbon as Metal-Free Peroxymonosulfate Activator for Tetracycline Hydrochloride Degradation: Performance, Mechanism and Biotoxicity. *Chem. Eng. J.* **2022**, *440*, 135760, doi:10.1016/j.cej.2022.135760.
9. Hou, S.Y.; Hu, H.L.; Fu, Q.; Xiao, T.Y.; Xie, J.Q.; Chan, S.H.; He, M.J.; Miao, B.; Zhang, L. Undaria Pinnatifida (Wakame)-Derived Fe, N Co-Doped Graphene-like Hierarchical Porous Carbon as Highly Efficient Catalyst for Activation of Peroxymonosulfate (PMS) toward Degradation of Tetracycline (TC). *Sep. Purif. Technol.* **2024**, *333*, 125980, doi:10.1016/j.seppur.2023.125980.
